# Losses never sleep – The effect of tax loss offset on stock market returns during economic crises

**DOI:** 10.1007/s11573-022-01134-4

**Published:** 2023-01-28

**Authors:** Reinald Koch, Svea Holtmann, Henning Giese

**Affiliations:** 1 KU Eichstaett-Ingolstadt, Ingolstadt, Germany; 2 Department of Taxation, Accounting and Finance, Paderborn University, Paderborn, Germany; 3 KU Research Institute for Taxation, Ingolstadt, Germany

**Keywords:** Tax loss offset, Economic crisis, Firm performance, H25, G01

## Abstract

We analyze to what extent more generous tax loss offset regulations are associated with a weaker decline and stronger recovery of firm stock prices during economic crises. We argue that an unrestricted loss carryforward and, particularly, an unrestricted loss carryback provides firms with additional liquidity, which should lower the risk of bankruptcy and can be used for investment purposes. Our empirical findings document that (1) an unrestricted loss carryforward and an unrestricted loss carryback result in a weaker decline and more timely recovery of stock prices during the considered crises, (2) this effect is stronger in high-tax countries, and (3) this effect is also dependent upon pre-crisis profitability.

## Introduction

Existing literature has documented that tax regulations can help mitigate the effects of economic crises and help firms recover (see, e.g., Slemrod and Wilson (2009) as well as Hemmelgarn and Nicodème (2010)). The corporate tax may, in principle, function as an automatic stabilizer in such a way as to cushion the influence of an economic downturn on relevant macroeconomic indicators (Buettner and Fuest 2010; Devereux and Fuest 2009). Besides, short-term incentives, like enhanced depreciation or a temporary reduction of corporate tax rates, can stimulate firm investment. However, the asymmetric design of worldwide corporate tax systems, characterized by an immediate taxation of profits and a limited and delayed refund for tax losses, limits the effectiveness of these instruments (Devereux et al. 2020). Extending tax loss offset possibilities for firms is, therefore, a policy measure used widely by industrialized countries not only during the economic crisis resulting from the current COVID-19 pandemic but also during prior recessions (see in this respect also the OECD recommendation, OECD (2020)). Offsetting tax losses reduces future tax payments or leads to a refund of previously paid taxes and should, in general, help firms overcome liquidity problems in times of an economic downturn. Moreover, firms can use excess funds resulting from tax losses for investment purposes, which may help them recover more timely. However, whether more favorable loss offset regulations help firms mitigate the effects of an economic crisis has not been validated. Following this notion, we empirically test whether and to what extent more generous loss offset regulations positively affect the stock market returns of 2729 listed firms from 24 industrialized countries during the past two economic crises: the financial crisis beginning in 2008 and the COVID crisis starting in 2020.

We empirically analyze the effects of the main types of tax loss offset regulations, i.e., loss carryback, loss carryforward, and intragroup loss offset, on the stock price of listed multinational firms. We refer to the firm’s stock price since it is expected to reflect the stabilizing and stimulating effects of more generous loss offset regulations. In doing so, we test three different hypotheses. In general, loss offset regulations should help improve firm liquidity by allowing for a cash-effective refund or a reduction of future tax payments. We, therefore, expect a positive impact of more generous loss carryforward and loss carryback regulations on stock market returns (Hypothesis 1a). Intragroup loss offset, as granted by group tax regimes in several countries, requires the existence of tax profits and tax losses in different affiliates of the same multinational firm to become effective. We, therefore, expect this instrument to be less effective during economic crises (Hypothesis 1b). Since the tax relief from the use of tax losses depends positively on the corporate tax rate, we expect the effect described by Hypothesis 1a to be more pronounced in high-tax countries (Hypothesis 2). Comparing the effects of the two types of intertemporal loss offset regulations, we argue that loss carryback offers firms two particular advantages in crises. First, tax refunds for incurred losses are granted as early as possible, i.e., immediately in the loss year. Second, tax refunds are independent of the (uncertain) future profit situation, i.e., generating post-crisis profits is not required. We, therefore, expect the positive effect from a generous loss carryback regulation, on average, to be stronger than the positive effect from a generous loss carryforward regulation if multinational firms report pre-crisis profits. Since a loss carryback is ineffective in the case of tax losses in prior years, we expect the opposite to be the case for multinational firms with pre-crisis losses (Hypothesis 3).

Analyzing the implications of loss offset regulations during economic crises has its merits over doing the same analysis during non-crisis times. First, we expect a higher relevance and a higher awareness of investors and analysts for the tax treatment of losses. Second, losses from an economic crisis should come mainly unexpectedly and should not be anticipated in pre-crisis capital market prices. Third, there is only a small within-country variation of tax loss offset rules, which usually stems from reforms during economic crises. Focusing on these timeframes allows us also to analyze how market prices respond to these changes.

Our empirical findings support all three hypotheses and show effects that are also of economically relevant size. Firms that benefit from an unrestricted loss carryforward or unrestricted loss carryback show a more than two percentage points lower stock price decline during the crisis. Besides, the recovery period is more than 160 days shorter. The positive effect from loss carryback is even stronger the higher the applicable tax rate. While the effect of group tax regimes seems to be more sensitive to the particular variable definitions used, overall, our findings indicate that intra-group loss offset regimes have a smaller effect on our dependent variables. The results also document considerable within-country heterogeneity in this response. While we observe that firms with pre-crisis profits benefit particularly strongly from a generous loss carryback, the design of loss carryforward regulations is more important for firms with pre-crisis losses. We also find that firms with high beta and low R&D intensity benefit particularly strongly.

This study contributes to at least three bodies of literature. One strand of literature investigates, in general, the interdependencies between tax policy and economic crises. Keen et al. (2010), e.g., analyze how tax policy impacts the emergence of economic crises, whereas Slemrod and Wilson (2009), as well as Hemmelgarn and Nicodème (2010), investigate how tax policy may help to overcome crises. A second strand examines, more specifically, the effect of loss offset regulations. These papers focus primarily on the impact of loss offset regulations on corporate investment decisions (e.g., Auerbach and Poterba 1987; Dreßler and Overesch 2013; Orihara 2015; Bethmann et al. 2018) or firm risk-taking (e.g., Langenmayr and Lester (2018) and Koch and Prassel (2011)). A third strand of literature analyzes, in general, the effect of tax policy on market valuation (e.g., Downs and Tehranian (1988) and Cutler (1988)). However, to the best of our knowledge, no existing study directly investigates the relevance of generous loss offset for the stock market development during an economic crisis. Focusing our analysis on stock market returns, we can observe how loss offset rules affect firms and to what extent investors reflect these effects in market values. Given the increasing importance of stock market returns for private consumption in recent years, developing a more stable stock market may also help prevent the crisis’s deterioration through this channel (see Auerbach and Feenberg (2000) in this respect). Besides the stabilizing effects of a generous loss offset for firm development during crises, our results also clearly document that investors consider the (complex) effects of intertemporal loss offset. This is an important finding with policy implications since a stable stock market development may help stabilize consumption (and thus the overall development of the economy) during an economic crisis.

However, we acknowledge that our study bears potential limitations, particularly resulting from our data structure. The cross-sectional nature of our data weakens any causal identification. We, e.g., cannot control for other policy interventions that aim to recover the economy during a crisis and are potentially correlated with tax loss offset rules. Moreover, unobserved country characteristics might (partly) explain our results. We address these concerns by presenting a comprehensive set of robustness tests for all three hypotheses, such as modifying our sample, conducting regression analyses that exploit within-country heterogeneity, using matched samples, and synthetic control groups. Based on the results presented in this paper, we are confident that the observed effects cannot be traced to such influences. However, being conservative, we do not try to establish causality but present evidence that the generosity of tax loss offset rules affects stock market performance.

The remainder of this paper proceeds as follows. The following section presents prior literature and highlights our contribution. Section 3 describes the institutional setting and derives our hypotheses. Section 4 introduces our empirical strategy. Section 5 outlines our data and discusses descriptive statistics. Section 6 provides our main results and several robustness tests. Section 7 concludes.

## Related literature

This paper builds on at least three bodies of literature.

A first strand of literature addresses the general role of tax policy in stabilizing firms during economic crises. To this end, Devereux et al. (2020) subdivide the current crisis into three phases ((1) an acute overall disruption, (2) an initial recovery phase, and (3) the longer term) and discuss the effectiveness of possible tax policy measures for these different phases. The authors argue that the asymmetric nature of the corporate tax may limit the effect of a more favorable corporate tax system (e.g., by reducing the tax rate or allowing an accelerated depreciation) for loss-making firms (see also Zwick and Mahon (2017)). Keen et al. (2010), as well as Hemmelgarn and Nicodème (2010), argue that the tax advantages from debt finance encouraged excessive use of debt finance, which contributed to the emergence of the 2008 financial crisis. Other studies point to the particular relevance of loss carryover regulations. These studies have documented that, besides the individual income tax, the corporate tax can generally function as an automatic stabilizer, particularly by stabilizing firm investment (Auerbach and Feenberg 2000). Lower corporate tax payments may improve firm liquidity and thus help to stabilize investment activities of financially constrained firms by reducing the volatility in net corporate earnings. However, if firms are in loss situations, this effect should be moderated by the tax treatment of losses (Auerbach and Feenberg 2000; Devereux and Fuest 2009; Buettner and Fuest 2010). Devereux and Fuest (2009) argue that financially constrained firms are often in loss situations and that the asymmetric design of the corporate tax makes it an ineffective automatic stabilizer. Based on a German set of firm data, Buettner and Fuest (2010) empirically document that only 20 percent of firms with capital market restrictions report positive taxable income. These studies thus clearly point to the advantages of a loss carryback, which provides firms with immediate cash advantage in loss situations. Dobridge (2021) empirically investigates the investment and stabilization effects of tax refunds for US firms during two recent recessions. She finds that US firms used 40 percent of received tax refunds for investment purposes in 2002, whereas in and after 2008, firms used tax refunds primarily to improve firm liquidity. Still, the policy measure led to a lower bankruptcy risk and a lower risk of a credit downrating. Zwick and Mahon (2017) analyze the effect of temporary tax incentives from bonus depreciation on firm investment. They find, amongst others, that firms respond strongly if the policy measure generates immediate cash advantages, whereas reactions are considerably smaller if cash effects only come in the future. This, again, points to the cross-effects between the availability of loss carryback and other profit tax incentives.

A second strand of literature investigates the relevance of tax losses and tax loss offset regulations in more detail, focusing also on non-crisis situations. Altshuler et al. (2009) and Henry and Sansing (2018) point to the high and increasing number of loss firms and, therefore, to the increasing relevance of tax losses. Using a comprehensive sample of US corporate tax returns for the period 1982–2005, Altshuler et al. (2009) show that the ratio of losses to positive income was much higher around the recession of 2001 than in earlier recessions. Henry and Sansing (2018) develop a new measure for tax avoidance and show that the established practice of dropping loss observations may considerably bias inferences about tax avoidance. More recent studies by Drake et al. (2020) and Schwab et al. (2022) show that valuation allowances related to prior-year losses instead of international tax avoidance drive significant parts of the variation in effective tax rates.

Tax losses and the treatment of tax losses influence different core business decisions. Several studies have investigated the interaction between the usability of tax losses and firm investment. Bethmann et al. (2018) show that firms reinvest one-third of tax refunds stemming from loss carrybacks, whereas they use the remainder to improve firm liquidity or return it to shareholders. However, a later market exit of low-productive loss-making firms induced by tax refunds may result in a misallocation of tax revenues. Using a panel of German Outbound FDI, Dreßler and Overesch (2013) show that a short carryforward period lowers investment, particularly for firms with a high loss probability. Other studies show that the asymmetric design of the corporate tax, i.e., the immediate taxation of profits on the one hand and the delayed offset possibilities for tax losses, may limit the effectiveness of other investment incentives. Edgerton (2010) analyzes the effectiveness of bonus depreciation on firm investment and shows that the asymmetric design of the tax system reduces the effectiveness of this instrument by four percent. Since the cash-flow situation largely drives the effectiveness, he predicts that such tax incentives have the smallest impact in an economic crisis.

According to Langenmayr and Lester (2018), risk-taking is also positively related to the length of the loss carryforward period. The tax rate positively affects risk-taking for firms that expect to use losses and has a weak negative effect for those that cannot. Other studies (Gamm et al. 2018; Simone et al. 2017; Hopland et al. 2018) look at the influence of tax losses on international profit shifting and document the existence of a shift-to-loss effect, i.e., a shifting of profits to foreign subsidiaries with low marginal tax rates for the reason of tax losses. Concerning financing decisions, Graham (1996) shows that the use of debt finance is negatively related to unused tax loss carryforwards.

A third strand of literature investigates the relationship between corporate tax rates, tax avoidance, and firm value. According to Modigliani and Miller (1958), the market value of a firm depends on the expected future net-of-tax profits. Lower expected future tax payments, as a consequence of, e.g., lower corporate tax rates, the application of tax avoidance practices, or efficient use of tax losses, should, therefore, be associated with an increase in firm value. The validity of this relationship has been tested empirically on various occasions. Literature has shown that more aggressive tax avoidance is not necessarily associated with an increase in firm values but that this relationship also depends on the firm’s governance (Desai and Dharmapala 2006), the uncertainty of future benefits from tax avoidance (Jacob and Schütt 2020), and reputational costs (Hanlon and Slemrod 2009; Huesecken et al. 2018). Contrastingly, studies that take corporate tax reforms as a natural experiment find the expected effect on firm market value. In this respect, several studies have, e.g., documented that US firms that were expected to benefit from the 2017 Tax Cuts and Jobs Act showed a positive stock market response around its enactment (see Diercks et al. 2020; Kalcheva et al. 2020; Wagner et al. 2018).

## Institutional setting & hypotheses

In all countries we consider in this paper, an asymmetric treatment of taxable profits and losses characterizes the corporate tax regime. Whereas profits are subject to immediate taxation, losses can become tax-effective only through a loss carryforward or—in some countries—also a loss carryback, which implies a time delay. Many countries further restrict these options for intertemporal loss offset concerning either the amount and/or time. Table 1 shows the availability of an unrestricted (amount and time) loss carryforward and unrestricted (amount) loss carryback at the beginning of the 2008 and 2020 economic crises in our sample countries. Tables 14 and 15 in the Appendix display country-specific regulations.

A further restriction to the use of tax losses within multinational firms stems from the separate entity principle, a common feature of the corporate tax system of all considered countries. According to the separate entity principle, the tax system treats all affiliates of a multinational group as separate tax subjects. As a result, a company cannot offset one subsidiary’s tax losses with another subsidiary’s taxable profits. Various countries offer specific group tax regimes allowing for an intra-group loss offset to prevent tax disadvantages from applying the separate entity principle. In most cases, however, these group tax regimes do not ensure a complete offset of profits and losses within a corporate group: First, the application of group tax regimes is subject to restrictive criteria in some countries (e.g., the necessity of a profit and loss transfer agreement in Germany). Second, in almost all countries, group tax regimes are restricted to domestic subsidiaries. A cross-border loss offset is allowed only in very few countries. Table 1 reports the number of countries in our sample that offered a group tax regime at the beginning of the two considered crises. Table 14 in the Appendix gives per-country information.

**Table 1:** Overview of intertemporal loss offset rules and group tax regimes

	LossCarryback		LossCarryforward		GroupTaxSystem	
	2007	2019	2007	2019	2007	2019
Yes	5	4	6	4	12	12
No	19	20	18	20	12	12

This table displays the distribution of loss carryback, loss carryforward, and group tax system rules in the 24 sample countries across the two crises. Data from the EY Worldwide Corporate Tax Guides

We argue that generous tax loss offset regulations may contribute significantly to stabilizing firms during the acute phase of an economic crisis and help firms to recover timely (see Devereux et al. (2020)). Following the separate entity principle, foreign affiliates of a multinational firm are subject to corporate tax in their country of residence. Consequently, a multinational firm should not only be affected by the tax regime in its home country but also by the regulations prevailing in the residence countries of its foreign affiliates. We, thus, cannot exclude that our findings underestimate the true effect of loss carryover regulations. However, we know from prior literature that the fraction of foreign income to total income is, on average, only around 20 percent (see Gaertner et al. (2021); Dyreng and Lindsey (2009)). Therefore, we expect this effect not to be substantial.^1^

To derive our hypotheses, we start by forming our expectations regarding the effect of more generous loss offset regulations on a firm’s downturn and recovery in an economic crisis. Using tax losses through a loss carryback or loss carryforward provides firms with additional liquidity, which may protect firms under financial constraints from bankruptcy and/or a credit downrating (see Dobridge (2021)). These cash inflows may exercise a stabilizing effect, particularly if firms are subject to financial constraints and taxable losses simultaneously. According to the results by Buettner and Fuest (2010), capital market restrictions frequently coincide with taxable losses in their sample of German multinationals in 80 percent of all cases.

Rapid use of tax losses or the expectation of rapid use of tax losses may also help firms recover more promptly from the crisis, particularly by fostering investment expenditure. Three mechanisms may explain this effect. First, firms may use liquidity resulting from tax losses for investments. Dobridge (2021) finds that US firms used 40 percent of tax refunds received at the end of the 2004 recession. In contrast, this ratio amounts to 30 percent in non-crisis situations, according to results reported by Bethmann et al. (2018). Second, unused tax losses put firms in a position of temporary tax exhaustion. In this situation, they do not benefit or benefit less from temporary fiscal stimulus, like temporary tax rate reductions or bonus depreciation, which governments frequently grant to mitigate the effects of an economic crisis. Third, the tax exhaustion status of the firms itself may constitute an investment incentive if firms can expect that future profits resulting from that investment are not subject to tax but can instead be used to offset prior losses. This, however, requires that tax loss carryforwards are not subject to severe restrictions concerning either time or amount.

Following the efficient market hypothesis (Fama 1970), we expect both the lower risk of bankruptcy and the improved expectations for the firm’s future development to be reflected in market values.^2^ In doing so, we implicitly assume that investors reflect the complex implications of tax loss offset regulations in their economic decisions. Extant literature has documented capital market responses to the introduction of new disclosure requirements for (complex) tax information (see, e.g., Dutt et al. 2019; Johannesen and Larsen 2016; Chen 2017; Hoopes et al. 2018).^3^ We assume that the same also holds for information on tax loss offset that listed firms have to provide in the interim and annual reports. We also believe that analysts and investors are particularly aware of these regulations in times of a general economic downturn, where many firms suffer from losses and many countries change these regulations.^4^ These considerations lead us to formulate our first hypothesis.

### Hypothesis 1a

*Multinational firms that are resident in countries with an unrestricted loss carryback and/or unrestricted loss carryforward show a weaker decline in market value during the acute phase of an economic crisis as well as a stronger and more timely recovery.*

Similar arguments can also be brought forward in favor of group taxation regimes. Also, the availability of an intragroup loss offset can result in an immediate utilization of tax losses and, thus, provide the same advantages as an immediate loss carryback. As outlined above, group tax regimes may be subject to restrictive application requirements and are usually limited in their application to domestic subsidiaries, which may reduce their effectiveness for multinational firms. Even more importantly, however, benefiting from an intragroup loss offset requires that multinational firms have profitable and unprofitable affiliates at the same time. We argue that an economic crisis with a global demand and/or supply shock will, in many cases, affect different sectors and regions within a multinational firm simultaneously, which should even more limit the effectiveness of a group tax regime. This leads us to formulate Hypothesis 1b as follows.

### Hypothesis 1b

*The availability of a group tax regime in the home country of a multinational firm is not associated with a weaker decline in market value during the acute phase of an economic crisis, as well as a stronger and more timely recovery.*

The size of all effects discussed in formulating Hypothesis 1a depends on the statutory corporate tax rate. Regardless of whether the corporate tax itself functions as an automatic stabilizer, the positive liquidity effects resulting from the use of tax losses are positively associated with a higher corporate tax rate. The same holds for the investment incentive effects that depend on an immediate use or -at least -an expected use of tax losses. We, therefore, expect the effects described by Hypothesis 1a to be stronger in countries featuring a higher corporate tax rate and formulate Hypothesis 2.

### Hypothesis 2

*The effects described by Hypothesis 1a are more pronounced in countries with a higher corporate tax rate.*

The effectiveness of loss offset regulations should also depend on firm characteristics, particularly the profit situation of the firm. A loss carryback regulation requires taxable profits within the carryback period to provide tax advantages. Therefore, we expect an unrestricted loss carryforward to be more effective than an unrestricted loss carryback for multinational firms with pre-crisis losses. In contrast, multinational firms with pre-crisis profits should benefit more from an unrestricted loss carryback. First, a loss carryback offers immediate tax relief and thus cash advantages earlier than a loss carryforward. Second, tax advantages from a loss carryforward are contingent on the availability of future profits. We, therefore, formulate Hypothesis 3 as follows.

### Hypothesis 3

*An unrestricted loss carryforward is more effective for multinational firms with pre-crisis losses, whereas multinational firms with pre-crisis profits benefit more from an unrestricted loss carryback.*

## Empirical strategy

**Fig. 1:**
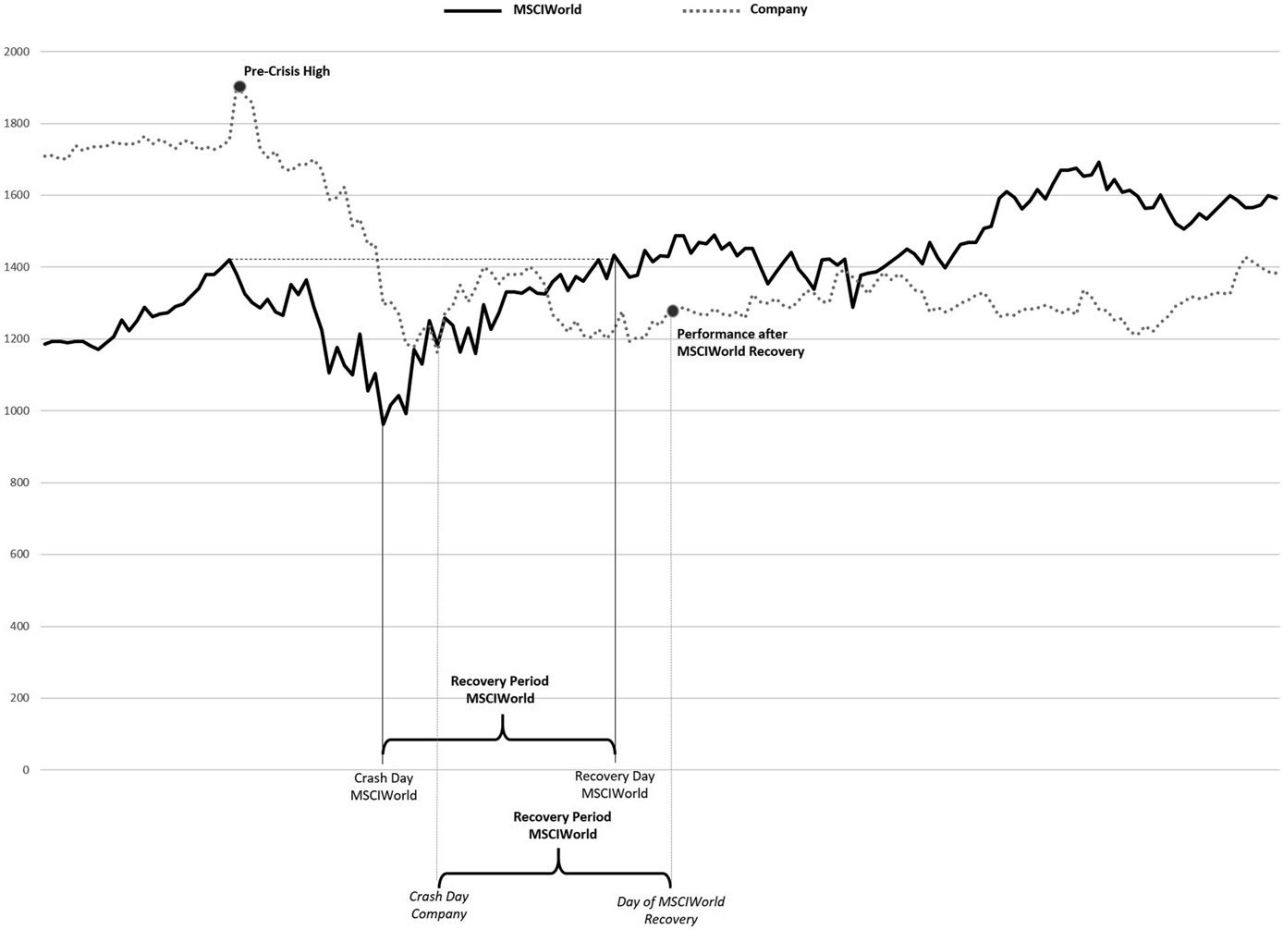
ReturnRecovery -Variable explanation

To test these hypotheses, we employ the following regression design for firm *i* and country *j* in crisis *t*.

1 $$\begin{aligned} \begin{aligned} \text {Performance}_{i,t}&= \beta _0 + \beta _1 \text {LossCarryback}_{j,t} + \beta _2 \text {LossCarryforward}_{j,t}\\&+ \beta _3 \text {GroupTaxSystem}_{j,t} + \delta X_{i,t} + \gamma Z_{j,t} + \mu _{\text {Industry}} + \eta _{\text {Crisis}} + \epsilon _{i,t} \end{aligned} \end{aligned}$$

where *Performance* is either measuring the share price decline of firm *i* during the acute phase of the respective crisis (referred to as crisis period in the following) or the recovery of firm *i*’s stock price afterward. The sample periods for our two considered recessions are August 2007 to May 2012 and January 2020 to April 2021 and are determined in line with extant literature and official publications.^5^ To measure the decline in firm *i*’s stock price (*ReturnDecline*), we use the percentage share price decline during the crisis period, calculated as the difference between the minimum stock price during the crisis and the maximum stock price reached before the crisis (adjusted for dividend distributions). We use two dependent variables to measure firm *i*’s stock price recovery from the crisis. The first recovery variable (*ReturnRecovery*) is determined by the following three-step procedure. We start by counting the number of days the MSCIWorld needed to recover from its lowest value during the respective crisis to reach its pre-crisis maximum. Next, we add this number of days to the firm-specific minimum stock price date during the respective crisis. Lastly, we calculate the recovery return for each firm by comparing the stock price of this day to firm *i*’s pre-crisis maximum (see Fig. 1 for an illustrative example). Values above one for this variable indicate that firms have fully recovered and are traded above the pre-crisis level. To determine the second recovery variable (*DaysRecovery*), we calculate the firm-specific time period (in days) between the day of the minimum stock price during the respective crisis period and the day at which the stock price reaches its pre-crisis maximum. The definition of these two variables captures both the strength and the timeliness of the stock price recovery.

*LossCarryback* is an indicator variable taking the value of 1 if country *j* offers a loss carryback of at least one year that is not restricted in amount. *LossCarryforward* takes the value of 1 if country *j* allows for a loss carryforward that is restricted neither in terms of time nor amount.^6^ According to Hypothesis 1a, we expect a positive and statistically significant coefficient estimate for $\beta _1$ and $\beta _2$ when explaining *ReturnDecline* and *ReturnRecovery*, indicating a smaller decline as well as a stronger recovery of stocks in countries with more generous loss offset provisions. When explaining *DaysRecovery*, we expect a significant and negative coefficient estimate for $\beta _1$ and $\beta _2$. Prior literature has identified group tax systems as an instrument to utilize tax benefits resulting from loss-making subsidiaries effectively (Oestreicher and Koch 2010; Rünger 2019). We, therefore, include an indicator variable (*GroupTaxSystem*) taking the value of 1 if a group tax system is in place and zero otherwise. According to Hypothesis 1b, however, we expect no significant effect of *GroupTaxSystem* during an economic crisis. We report the values for our three loss offset variables country-wise in Appendix Table 15.

*X* represents a vector of firm control variables. We control for firm size (*Size*) measured by total assets and *Leverage* measured by total debt to total assets. Since *Size* is highly skewed, we include it in terms of its natural logarithm. Furthermore, we include the firm-specific risk (*Beta*). We use lagged values for all firm controls since current year balance sheet information is published with a time lag and can, therefore, not be reflected in current market prices.

*Z* represents a vector of country control variables to account for general differences across the sample countries. We include the statutory tax rate (*TaxRate*), the unemployment rate (*Unemployment*), GDP per Capita (*GDPperCapita*), GDP growth (*GDPGrowth*), country risk (*CountryRisk*), the population (*Population*), the US dollar exchange rate (*ExchangeRate*), and the inflation rate (*Inflation*). We include *GDPperCapita* in terms of its natural logarithm and, again, all controls in terms of their one-year lags. Besides, *ChangeUnemployment* and *ChangeGDPperCapita* control for economic development during the respective crisis period.

We use industry and crisis fixed effects to control for industry and crisis-specific properties. Furthermore, we use Driscoll-Kraay standard errors to account for serial correlation between the crises (Driscoll and Kraay 1998; Hoechle 2007) and perform multi-way clustering on year-industry-level and country-level. We weight each observation as to assign equal weights across countries and crises. This approach should ensure that our results are not biased by differences in the size of stock indices across countries^7^ and differences in the availability of data across crises.^8^

According to Hypothesis 2, we expect the positive effect of generous loss offset rules to increase with the statutory corporate tax rate.^9^ To test this relation, we modify Eq. 1 by adding an interaction term of the loss carryback dummy and the statutory tax rate, as well as the loss carryforward dummy and the statutory tax rate, leading to the following equation. We expect the coefficient estimate of the interaction terms to be statistically significant and positive for *ReturnDecline* and *ReturnRecovery* and negative for *DaysRecovery*.

2 $$\begin{aligned} \begin{aligned} \text {Performance}_{i,t}&= \beta _0 + \beta _1 \text {LossCarryback}_{j,t} * \text {TaxRate}_{j,t} +\beta _2 \text {LossCarryback}_{j,t} \\&+ \beta _3 \text {LossCarryforward}_{j,t} * \text {TaxRate}_{j,t} + \beta _4 \text {LossCarryforward}_{j,t} \\&+ \beta _5 \text {GroupTaxSystem}_{j,t} + \beta _6 \text {TaxRate}_{j,t} + \delta X_{i,t} + \gamma Z_{j,t} \\&+ \mu _{\text {Industry}} + \eta _{\text {Crisis}} + \epsilon _{i,t} \end{aligned} \end{aligned}$$

Hypothesis 3 predicts that firms with pre-crisis tax losses are affected more by a generous loss carryforward. In contrast, loss carryback regulations should be of greater relevance for firms with pre-crisis tax profits. We test this hypothesis by splitting the sample based on the accounting profits in the pre-crisis year.^10^ We then run the baseline regression (Eq. 1) for both subsamples and compare the coefficients estimated for *LossCarryback* and *LossCarryforward* in each specification.

## Data and descriptive analysis

To test our hypotheses, we examine companies listed in the benchmark index of 24 OECD and EU countries with a population of more than 10 million.^11^ We have several reasons for this selection. First, the considered countries represent approximately 80 percent of global economic activity (World Bank 2021), and firms listed in the respective benchmark indices represent large shares of the overall free-float market capitalization in the respective countries. Second, the capital markets of these countries are well developed, making it more likely that investors are capable of reflecting the complex loss offset regulations correctly in market prices. Additionally, we restrict our sample to companies with positive pre-crisis returns and negative returns during the crisis.

For each firm in our sample, we obtain balance sheet information and daily stock market return data from 2007 to 2021 using Thomson Reuters. We complement these data by hand-collected information on the country’s tax loss regulations, i.e., the availability and details of a loss carryforward, a loss carryback, and an intragroup loss offset. We take this tax information from the EY Worldwide Corporate Tax Guides.^12^ Further, we use information on statutory corporate tax rates from KPMG and additional country controls from the International Monetary Fund. Appendix Table 16 presents descriptive statistics for our sample.

According to Appendix Table 16, firms in our sample experienced, on average, a 49 percent stock price decline during the two crises, while the firms recovered, on average, to 111 percent of the pre-crisis level. Only 18 (17) percent of our sample firms are located in countries with an unrestricted loss carryback (carryforward), while 27 percent can benefit from a group tax system to offset losses across affiliates. We also provide a correlation matrix for all explanatory variables included in the baseline regression (see Table 17 in the Appendix). While *LossCarryback* and *LossCarryforward* are weakly negatively correlated (– 0.06), we observe positive correlations between *LossCarryforward*, *GroupTaxSystem*, and *TaxRate* of between 0.3 and 0.5.^13^

In Table 2, we descriptively compare the share of firms for which the stock price fully recovers within our sample period, depending on the availability of an unrestricted loss carryback or unrestricted loss carryforward. We find that firms from countries with unrestricted loss carryback have a firm share with a full recovery that is nine percentage points higher compared to all other firms. This difference is also statistically significant on the 1%-level. Contrary, we find no statistical difference for *LossCarryforward*.

**Table 2:** Full recovery rates depending on loss offset possibilities

	LossCarryback	LossCarryforward
Unrestricted	75.31%	65.37%
Restricted or not available	66.06%	68.22%
Diff	9.25%	− 2.85%
F−Value	0.0000***	0.1978

This table presents the share of recovered firms in countries with an unrestricted *LossCarryback* (*LossCarryforward*) compared to the recovery rate of firms in countries without an unrestricted *LossCarryback* (*LossCarryforward*). E.g., 75.31% represents the recovery rate for firms with unrestricted loss carryback possibility, whereas 66.06% represents the recovery rate for firms with restricted (or no) loss carryback option. Own calculations. ***labels statistical significance at 1% level

In Appendix Table 18, we provide further descriptives for the sub-samples of countries belonging to the unrestricted loss carryback (carryforward) group or not.

## Empirical results

### Baseline results

#### General effects of a generous loss offset (Hypothesis 1)

According to Hypothesis 1a, we expect firms from countries with unrestricted loss carryforward and loss carryback to perform better in terms of stock price development than firms from countries with more restrictive loss offset regulations. We expect no similar effect for countries that offer an intra-group loss offset (Hypothesis 1b). Regression results for Hypothesis 1a and Hypothesis 1b are presented in Table 3. To measure the firm’s stock price performance during the crisis, we use the percentage decline of the firm’s share price, calculated as the difference between the minimum stock price during and the maximum stock price prior to the crisis (*ReturnDecline*, Column 1), the recovery of the stock price during the interval the MSCIWorld recovered from the respective decline (*ReturnRecovery*, Column 2) and the number of days between the crisis minimum and the full recovery to the pre-crisis maximum (*DaysRecovery*^14^, Column 3). We use indicator variables for an unrestricted loss carryback of at least one year (*LossCarryback*), an unrestricted loss carryforward (*LossCarryforward*), and the availability of a group tax system (*GroupTaxSystem*).

Supporting Hypothesis 1a, we find positive and statistically significant coefficient estimates for *LossCarryback* and *LossCarryforward* in all three columns. An unrestricted *LossCarryback* (*LossCarryforward*) mitigates the share price decline by 2.5 (2.6) percentage points. This effect is equivalent to 5.1 (5.3) percent of the average crisis decline in our sample (49 percent). According to our results, the effect of a generous loss offset is even more pronounced when we turn to our two recovery variables. Stock prices of firms located in countries with an unrestricted *LossCarryback* (*LossCarryforward*) experience, on average, a 23 (10) percentage points larger recovery and recover to their pre-crisis maximum 179 (167) days earlier. These effects are equivalent to 20.7 (9.0) and 50.4 (47.0) percent of the sample means. Besides, our results in Table 3 indicate no clear dominance of either of the two loss offset regulations. Whereas in specification (1), *LossCarryforward* exercises a larger effect than *LossCarryback*, the opposite can be observed for the recovery variables in specifications (2) and (3).^15^

In Hypothesis 1b, we hypothesize that a group tax system is not similarly effective during a crisis, hence, expecting an insignificant coefficient estimate for the *GroupTaxSystem* variable. Supporting Hypothesis 1b, the coefficient estimate for *GroupTaxSystem* is indistinguishable from zero at conventional levels in specifications (1) and (2), besides being negative or small in magnitude. Only in Specification (3) we find a significantly negative effect indicating that the availability of a group tax system reduces the recovery period. However, the significance level and the effect size are smaller than for the other two loss offset variables.

**Table 3:** Baseline results

Dependent Variable	ReturnDecline	ReturnRecovery	DaysRecovery
	(1)	(2)	(3)
LossCarryback	0.0250**	0.2464***	− 179.4676**
	(1.97)	(5.34)	(− 2.52)
LossCarryforward	0.0257*	0.0968*	− 167.1551**
	(1.75)	(1.88)	(− 2.35)
GroupTaxSystem	− 0.0014	0.0351	− 106.7540*
	(− 0.12)	(0.71)	(− 1.90)
TaxRate	− 0.5146***	0.1848	922.5706**
	(− 4.00)	(0.34)	(2.03)
Unemployment	− 0.0005	− 0.0060	− 1.2670
	(− 0.39)	(− 1.04)	(− 0.32)
GDPperCapita	− 0.0055	− 0.0396	26.5228
	(− 0.32)	(− 0.56)	(0.50)
Inflation	0.0047***	0.0245***	− 18.2526***
	(2.85)	(3.68)	(− 3.61)
GDPGrowth	− 0.0148**	− 0.0205	− 2.0944
	(− 2.33)	(− 0.74)	(− 0.08)
ExchangeRate	− 0.0000	0.0001*	− 0.0412
	(− 0.12)	(1.74)	(− 1.55)
CountryRisk	0.0060**	− 0.0154	− 3.8555
	(2.35)	(− 1.51)	(− 0.50)
Population	0.0001**	− 0.0000	− 0.1003
	(2.45)	(− 0.42)	(− 0.89)
Beta	− 0.0884***	− 0.1211**	47.6735
	(− 7.59)	(− 2.54)	(1.10)
Leverage	− 0.0065***	− 0.0217***	− 65.5279
	(− 3.52)	(− 2.40)	(− 0.51)
Size	0.0043	− 0.0228	33.8179*
	(0.81)	(− 1.03)	(1.73)
ReturnPreCrisis	− 0.0139***	− 0.0316	13.2701
	(− 2.73)	(− 1.03)	(0.77)
ChangeUnemployment	− 0.0280	− 0.2454***	138.1258
	(− 1.26)	(− 2.75)	(1.47)
ChangeGDPperCapita	0.0664	0.7403**	557.8078**
	(1.26)	(2.36)	(2.17)
Crisis & Industry FE	Yes	Yes	Yes
Observations	3139	2729	2126
Centered R^2^	0.1346	0.0759	0.1030

This table represents the baseline results for Hypotheses 1a and 1b. Data from the International Monetary Fund, EY Worldwide Corporate Tax Guide, and Thomson Reuters. The observational units are firms. See Appendix Table 16 for variable definitions. Heteroskedasticity-robust Driscoll-Kraay standard errors with multi-way clustering on year-industry-level and country-level in parentheses. *Indicates significance at the 10% level, **indicates significance at the 5% level, ***indicates significance at the 1% level

Prior literature has pointed to the stabilizing effect of the corporate income tax during a crisis (Auerbach and Feenberg 2000; Buettner and Fuest 2010). Given that the corporate income tax absorbs part of the overall income, it partially neutralizes reduced firm output. Therefore, higher tax rates could potentially be associated with smaller stock price declines and faster recoveries. However, the coefficient estimate for *TaxRate* is negative (positive) and statistically significant in Column 1 (3), indicating that a higher corporate tax rate is associated with a stronger decline during the downturn and a slower recovery. A one standard deviation higher *TaxRate* enlarges the stock price decline by two percentage points. This result supports findings by Devereux and Fuest (2009), who report only a marginal stabilizing effect of the corporate income tax for the UK. In accordance with prior literature, we expect higher values for firm *Beta* to be associated with a stronger decline (weaker recovery) (Levy and Galili 2006; Luo et al. 2020; Wang and Young 2020). Supporting this expectation, we find statistically significant coefficient estimates in line with this prediction in two out of three specifications.

Classifying loss offset rules as being relevant or irrelevant solely based on no restrictions regarding time and amount, as done for our baseline regressions reported in Table 3, is not free of ambiguity. While the use of separate dummies for loss carryforward and loss carryback enables us to directly compare the effects of different loss offset regulations, we disregard, to a certain extent, existing variation in these rules (e.g., we regard a loss carryback with amount restriction as equivalent to no loss carryback). Besides, incorporating separate dummies in the same regression may suffer from a correlation between these variables. In order to fully exploit the existing variation in loss offset regulations and test the robustness of our findings against alternative definitions of our loss offset variables, we use two additional variables that evaluate the restrictiveness of loss offset rules based on a combined scoring model.

*Combined1*, used in Table 4, can take the values 0, 1, 2, 3, and 4 and is included in terms of separate categorical variables. The score is increased by one point for a loss carryback of at least one year and a loss carryforward without time restriction. One further point is added for each of these regulations not being restricted in amount. We consider the different score levels as separate categorical variables in order to be able to make the effect of different score levels transparent and test them for statistical significance. Our results in Table 4 indicate consistently that higher values for *Combined1* are associated with a weaker decline and a stronger and faster recovery of stock prices. For all three specifications, the effect size for *Combined1=3* and *Combined1=4* exceeds the effect size for the next smaller score. Moreover, seven out of nine of these coefficient estimates are statistically significant. F-tests for the significance of the differences in coefficient estimates reported in the lower section of the table reveal statistical significance in eight out of nine cases.

*Combined2* further differentiates depending on time-related restrictions. Whereas again, one point is granted for loss carryback or loss carryforward without amount-related restriction, values between zero and one are added to the score depending on the loss carryforward or loss carryback period.^16^ Accordingly, *Combined2* can take values out of the real interval from zero to four, including the boundaries. Since this variable is not restricted to integer values, we include the score as a metric variable. We report regression results using *Combined2* to assess loss offset possibilities in Table 19 in the Appendix. Again, we find statistically significant effects for all three dependent variables, pointing in the expected direction.

While our findings suggest that regression results for Hypothesis 1a are not sensitive to the particular definition of loss offset variables, we observe a mixed picture with regard to Hypothesis 1b. While *GroupTaxSystem* has a weak effect in our baseline regressions (Table 3), we find statistically significant coefficient estimates for this variable that point in the expected direction in five of six specifications reported in Table 4 and Appendix Table 19.

**Table 4:** Heterogeneity in loss offset possibilities

Dependent Variable	ReturnDecline	ReturnRecovery	DaysRecovery
	(1)	(2)	(3)
Combined1			
2	0.0891***	0.2693**	− 66.2063
	(3.27)	(2.07)	(− 0.51)
3	0.0907***	0.3790***	− 207.9032
	(3.21)	(2.80)	(− 1.58)
4	0.2109***	0.8194***	− 499.7195**
	(5.12)	(4.84)	(− 2.47)
GroupTaxSystem	0.0316**	0.1062*	− 122.4253**
	(2.27)	(1.76)	(− 2.13)
TaxRate	− 0.3058***	0.2114	713.7221
	(− 2.75)	(0.47)	(1.44)
*F−Test*			
2=3	0.8983	0.0260**	0.0669*
2=4	0.0001***	0.0000***	0.0028***
3=4	0.0003***	0.0014***	0.0331**
Controls	Yes	Yes	Yes
Crisis & Industry FE	Yes	Yes	Yes
Observations	3139	2729	2126
Centered R^2^	0.1661	0.0799	0.1034

This table represents the heterogeneity analysis for *LossCarryback* and *LossCarryforward* time and amount restrictions. Data from the International Monetary Fund, EY Worldwide Corporate Tax Guide, and Thomson Reuters. The observational units are firms. *Combined1* is a score-based measure with values of 0, 1, 2, 3, and 4 and is included in terms of categorical variables. The score increases by one point for a loss carryback of at least one year as well as a loss carryforward without time restriction. One further point is added for each of these regulations not being restricted in amount. See Appendix Table 16 for the remaining variable definitions. Heteroskedasticity-robust Driscoll-Kraay standard errors with multi-way clustering on year-industry-level and country-level in parentheses. *Indicates significance at the 10% level, **indicates significance at the 5% level, ***indicates significance at the 1% level

Several countries reformed their tax loss offset rules in the two recessions considered in this paper. We further investigate the implications of such reforms by considering an additional explanatory variable, *IncreasedLossOffset*. This variable takes the value of 1 if a country has extended its loss offset regulations during the respective crisis in a way that *LossCarryback* or *LossCarryforward* changes from zero to one. We observed such reforms in two (six) of our sample countries during the 2008 (2020) crisis.^17^ Considering that the enhanced loss offset was not available during the entire crisis period, we expect, in theory, a positive effect on our two recovery variables and no significant effect on *ReturnDecline*. Our regression results reported in Table 5 predominantly support these expectations. While we find no significant effect of *IncreasedLossOffset* on *ReturnDecline*, *ReturnRecovery* is significantly higher in countries where loss offset has been extended during the crisis. The size of this effect is smaller than the coefficients estimated for *LossCarryback* and *LossCarryforward*, which is, again, consistent with an implementation during the crisis.

**Table 5:** Changes in loss offset regulations during crisis

Dependent Variable	ReturnDecline	ReturnRecovery	DaysRecovery
	(1)	(2)	(3)
IncreasedLossOffset	0.0102	0.0853*	– 22.1113
	(0.72)	(1.85)	(– 0.53)
LossCarryback	0.0264**	0.2587***	– 182.4403**
	(2.03)	(5.64)	(– 2.51)
LossCarryforward	0.0250*	0.0910*	– 165.6534**
	(1.68)	(1.76)	(– 2.36)
GroupTaxSystem	– 0.0007	0.0400	– 108.1561*
	(– 0.06)	(0.80)	(– 1.89)
TaxRate	– 0.5108***	0.2161	916.3873**
	(– 4.00)	(0.41)	(2.03)
Crisis & Industry FE	Yes	Yes	Yes
Observations	3129	2729	2126
Centered R^2^	0.1351	0.0770	0.1032

This table represents an additional test to our baseline specification, including an indicator variable capturing the extension of loss offset regulations during the crisis. *IncreasedLossOffset* takes the value of one if the respective home country increased its *LossCarryback* or *LossCarryforward* regulations during the course of the crisis. We only consider changes with unrestricted amounts. Data from the International Monetary Fund, EY Worldwide Corporate Tax Guide, and Thomson Reuters. The observational units are firms. See Appendix Table 16 for variable definitions. Heteroskedasticity-robust Driscoll-Kraay standard errors with multi-way clustering on year-industry-level and country-level in parentheses. *Indicates significance at the 10% level, **indicates significance at the 5% level, ***indicates significance at the 1% level

#### Generous loss offset and the tax rate (Hypothesis 2)

According to Hypothesis 2, the effect of a loss carryback should be more pronounced for firms in countries with high corporate tax rates. To test this prediction, we add two interaction terms of the statutory tax rate and (a) the *LossCarryback* variable and (b) the *LossCarryforward* variable to Eq. 1.

Table 6 reports the results. In line with our hypothesis, we find a positive and statistically significant effect for the interaction term of *LossCarryback* and *TaxRate* for *ReturnDecline* and a negative and significant effect for *DaysRecovery*. A one standard deviation increase in the statutory tax rate corresponds to a 3.5 percentage points smaller decline if the country offers an unrestricted loss carryback. We find no similar effect for the interaction term of *LossCarryforward* and *TaxRate*. The estimated coefficients are either insignificant (Columns (1) and (3)) or point in the direction opposite to our theoretical predictions (Column (2)). We have two explanations for this outcome. First, the benefits from the use of loss carryforwards are realized in future years and thus depend on future statutory tax rate rather than the current one. Although the current tax rate may generally be the best prediction of future ones, this relation may be weaker in times of an economic crisis, where corporate tax rates frequently change. Second, the statutory tax rate shows a sizeable correlation with *LossCarryforward*, making an accurate identification of interaction effects more challenging.

**Table 6:** Loss offset possibilities & tax rate

Dependent Variable	ReturnDecline	ReturnRecovery	DaysRecovery
	(1)	(2)	(3)
LossCarryback#TaxRate	0.8683***	0.7358	− 1,770.5696*
	(3.13)	(0.63)	(− 1.88)
LossCarryforward#TaxRate	− 0.3239	− 4.4468***	588.1357
	(− 0.99)	(− 3.67)	(0.49)
LossCarryback	− 0.1979**	0.0633	276.1198
	(− 2.60)	(0.20)	(1.02)
LossCarryforward	0.1179	1.3623***	− 333.7327
	(1.25)	(3.95)	(− 1.04)
GroupTaxSystem	− 0.0019	0.0077	− 106.5571*
	(− 0.15)	(0.14)	(− 1.82)
TaxRate	− 0.5946***	0.7053	1,090.1127**
	(− 3.68)	(0.96)	(2.01)
Crisis & Industry FE	Yes	Yes	Yes
Observations	3139	2729	2126
Centered R^2^	0.1447	0.0807	0.1078

This table represents the results for Hypothesis 2. Data from the International Monetary Fund, EY Worldwide Corporate Tax Guide, and Thomson Reuters. The observational units are firms. See Appendix Table 16 for variable definitions. Heteroskedasticity-robust Driscoll-Kraay standard errors with multi-way clustering on year-industry-level and country-level in parentheses. *Indicates significance at the 10% level, **indicates significance at the 5% level, ***indicates significance at the 1% level

#### Heterogenous effects for firms with pre-crisis profits and losses (Hypothesis 3)

The main advantage of a loss carryback compared to a loss carryforward is the immediate cash effect. However, companies only benefit from such regulation if they were profitable in pre-crisis years. Hence, we expect an unrestricted loss carryback to be more relevant for firms with pre-crisis tax profits than an unrestricted loss carryforward (Hypothesis 3). The opposite is expected for firms with pre-crisis tax losses. To test this prediction, we split our sample based on pre-crisis accounting profitability and estimate Eq. 1 for both sub-samples. Results are reported in Table 7. Columns 1, 3, and 5 (2, 4, and 6) display the estimates for companies with pre-crisis losses (profits). Comparing the difference in effect size for *LossCarryback* and *LossCarryforward* across the two groups shows - as expected - a more pronounced role of loss carryforward for loss-making firms, while a loss carryback is more relevant for firms with pre-crisis profits. In all six specifications, the coefficient estimate size differs in the expected direction, and the difference is statistically significant in half of the cases.^18^

**Table 7:** Pre-crisis profitability

Dependent Variable	ReturnDecline		ReturnRecovery		DaysRecovery	
	Loss	Profit	Loss	Profit	Loss	Profit
LossCarryback	0.1130*	0.0204*	0.5222**	0.2314***	138.0454	– 176.4486**
	(1.91)	(1.67)	(2.40)	(5.05)	(0.38)	(– 2.54)
LossCarryforward	0.22285***	0.0169	1.0381***	0.0791	–181.9776	–166.0012**
	(3.13)	(1.12)	(3.02)	(1.52)	(− 0.50)	(– 2.31)
GroupTaxSystem	0.0771	− 0.0061	– 0.4346	0.0408	– 87.4858	– 113.5435**
	(1.55)	(− 0.53)	(– 1.65)	(0.80)	(– 0.68)	(– 1.96)
TaxRate	– 1.0669**	– 0.5081***	– 0.7964	0.0347	841.2105	1068.7178**
	(− 2.02)	(− 3.95)	(− 0.33)	(0.06)	(0.65)	(2.32)
*F−Test*						
LCB=LCF	0.0656*	0.8379	0.0781*	0.0055***	0.1351	0.8078
Crisis & Industry FE	Yes	Yes	Yes	Yes	Yes	Yes
Observations	253	2,882	212	2,513	147	1,976
Centered R^2^	0.3238	0.1301	0.1379	0.0774	0.3016	0.1081

This table represents the results for Hypothesis 3. Data from the International Monetary Fund, EY Worldwide Corporate Tax Guide, and Thomson Reuters. The observational units are firms. See Appendix Table 16 for variable definitions. Heteroskedasticity-robust Driscoll-Kraay standard errors with multi-way clustering on year-industry-level and country-level in parentheses. *Indicates significance at the 10% level, **indicates significance at the 5% level, ***indicates significance at the 1% level

### Heterogeneity in response

In this section, we analyze the heterogeneity in the firm-level response to tax loss offset rules more closely. To this end, we split the sample into two subsamples depending on firm size (as measured by total assets), firm risk (as measured by firm beta), and R&D intensity (as measured by R&D expenditures). Our results for the two subsamples of firm size indicate that findings are weaker for the upper half of firm size. This finding may indicate that the stocks of these firms are, ceteris paribus, less volatile than the stocks of smaller firms (e.g., because large firms tend to be more diversified and are thus able to cushion cashflows across regions, branches, or business areas resulting in a lower cash flow volatility) or that these firms also have access to other tax planning strategies to effectively utilize tax losses making loss carryover regulations less important to them.

In the middle section of Table 8, we split the sample depending on firm beta. Our findings document that firms with an above-average risk benefit slightly more from an unrestricted loss carryback or loss carryforward. We additionally investigate the heterogeneous effect of generous loss offset rules for firms with above-average and below-average R&D intensity in the lower section of Table 8. Our results clearly and consistently document that loss offset regulations are particularly relevant for firms with low R&D intensity. Firms benefit most from a generous loss carryforward and loss carryback if they experience significant losses, which reverse in the short run. Our results may, thus, indicate that R&D activities are associated with a lower probability of losses and/or more persistent losses. Another possible explanation for the smaller impact of loss offset rules for high-R&D firms is the availability of preferential tax regimes for this type of investment. The availability of tax credits or a lower tax rate on R&D income may mitigate the relevance of other tax (base) regulations.

**Table 8:** Heterogeneity in response

Dependent Variable	Assets					
	ReturnDecline		ReturnRecovery		DaysRecovery	
	Low	High	Low	High	Low	High
LossCarryback	0.0446*	0.0173	0.1792**	− 0.0401	− 122.8995	− 60.1685
	(1.72)	(1.20)	(2.37)	(− 0.65)	(− 1.58)	(− 0.89)
LossCarryforward	0.0609**	0.0229	0.3290***	0.0565	− 236.5449**	− 86.4313
	(2.04)	(1.21)	(4.21)	(0.71)	(− 2.36)	(− 1.09)
GroupTaxSystem	− 0.0253	− 0.0212	− 0.0884*	− 0.0833	− 63.8781	− 96.8215
	(− 1.54)	(− 1.19)	(− 1.89)	(− 1.03)	(− 1.26)	(− 0.84)
TaxRate	− 0.3941	− 0.4896***	− 3.3080***	0.5293	1568.7256**	1528.2897**
	(− 1.01)	(− 2.72)	(− 4.40)	(0.67)	(2.20)	(2.14)
Controls	Yes	Yes	Yes	Yes	Yes	Yes
Crisis & Industry FE	Yes	Yes	Yes	Yes	Yes	Yes
Observations	1570	1569	1365	1364	1063	1062
Centered R^2^	0.1594	0.1392	0.1076	0.1450	0.1347	0.1403

Dependent Variable	Beta					
	ReturnDecline		ReturnRecovery		DaysRecovery	
	Low	High	Low	High	Low	High
LossCarryback	0.0167	0.0207	0.1827***	0.1976***	− 87.4853	− 201.4051
	(1.07)	(1.21)	(2.82)	(4.07)	(− 1.42)	(− 1.49)
LossCarryforward	0.0194	0.0298	0.0243	0.1055	− 103.8576*	− 192.3736
	(1.04)	(1.53)	(0.31)	(1.54)	(− 1.66)	(− 1.63)
GroupTaxSystem	0.0060	− 0.0212	0.0745	− 0.0330	− 101.5327**	− 142.4436
	(0.40)	(− 1.30)	(1.15)	(− 0.55)	(− 2.07)	(− 1.33)
TaxRate	− 0.4088**	− 0.6052***	0.8836	− 0.1646	983.0383*	767.7152
	(− 2.37)	(− 3.82)	(1.12)	(− 0.30)	(1.85)	(1.13)
Controls	Yes	Yes	Yes	Yes	Yes	Yes
Crisis & Industry FE	Yes	Yes	Yes	Yes	Yes	Yes
Observations	1,570	1,569	1,364	1,364	1,062	1,063
Centered R^2^	0.1011	0.1830	0.0887	0.0620	0.1362	0.0836

Dependent Variable	R & D					
	ReturnDecline		ReturnRecovery		DaysRecovery	
	Low	High	Low	High	Low	High
LossCarryback	0.0405**	0.0324	0.4193***	0.1041*	− 266.1202***	− 63.3288
	(2.10)	(1.57)	(5.40)	(1.78)	(− 3.00)	(− 1.03)
LossCarryforward	0.0342	0.0305	0.1913**	0.0353	− 191.7370**	− 90.8977
	(1.53)	(1.41)	(2.19)	(0.53)	(− 2.08)	(− 1.42)
GroupTaxSystem	− 0.0208	0.0238	0.0045	0.0205	− 69.0135	− 34.2041
	(− 1.40)	(0.96)	(0.08)	(0.22)	(− 1.43)	(− 0.54)
TaxRate	− 0.2605*	− 0.5039**	1.2967*	− 0.1934	236.1706	98.9844
	(− 1.69)	(− 2.20)	(1.75)	(− 0.28)	(0.49)	(0.15)
Controls	Yes	Yes	Yes	Yes	Yes	Yes
Crisis & Industry FE	Yes	Yes	Yes	Yes	Yes	Yes
Observations	1,300	1,301	1,117	1,118	882	882
Centered R^2^	0.1556	0.1813	0.0979	0.0886	0.1597	0.0699

This table represents the results of a heterogeneity analysis based on firms’ size, risk, and R&D expense. Observations are allocated into the low and high categories based on a median split. Data from the International Monetary Fund, EY Worldwide Corporate Tax Guide, and Thomson Reuters. The observational units are firms. See Appendix Table 16 for variable definitions. Heteroskedasticity-robust Driscoll-Kraay standard errors with multi-way clustering on year-industry-level and country-level in parentheses. *Indicates significance at the 10% level, **indicates significance at the 5% level, ***indicates significance at the 1% level

### Robustness

#### Variable definitions and composition of the sample

We perform several robustness tests to document the validity of our baseline findings for all three hypotheses and report the results below or in the Appendix.

A first set of robustness tests for Hypothesis 1 is reported in Table 9. Our baseline findings rely on a weighting of observations that assigns equal weights to all countries and both crises. This weighting follows the idea that our panel of firm data should not be biased towards countries with large stock indices (e.g., Japan) or years with better data availability. Nonetheless, we test whether our findings also hold for an unweighted panel. Respective results for Hypothesis 1 are reported in Columns (1) to (3) of Table 9 below. *LossCarryback* exercises a significant effect in the expected direction for all three dependent variables, whereas *LossCarryforward* mitigates both *ReturnDecline* and *DaysRecovery* in a statistically significant manner.

A second robustness test is based on the notion that countries are hit differently by economic crises, which may be associated with a different sensitivity of firms to tax loss offset regulations. In order to test to what extent our findings are driven by observations from countries in particular economic turmoil, we re-estimate our baseline regressions for a reduced sample, which disregards observations from countries that belong to the top decile of CountryRisk in the year of the crisis.^19^ We report respective results for Hypothesis 1 in Columns (4) to (6) of Table 9. Statistical inferences are now even stronger compared to our baseline regressions for both variables testing Hypothesis 1a.

We report the results of a third robustness test in Column (7) of Table 9. Here, we apply a modified definition of *DaysRecovery*. According to the original definition, we only consider those observations that actually reach their pre-crisis maximum within our sample period. To avoid any distortions resulting from this definition, we assign the value of 1,667 days to each observation if the stock did not fully recover before. This value is equivalent to the 99th percentile of this variable. This procedure also avoids any potential distortions from influential outliers. Again, the resulting coefficient estimates for *LossCarryback* and *LossCarryforward* are negative and statistically significant.

Robustness tests reported in Table 9 not only support previous findings regarding Hypothesis 1a, but they also give strong support for Hypothesis 1b. *GroupTaxSystem* exercises a significant effect in the expected direction in none of the seven specifications.

**Table 9:** Robustness tests I Hypothesis 1

Dependent Variable	Unweighted			Reduced Sample			Full Sample
	ReturnDecline	ReturnRecovery	DaysRecovery	ReturnDecline	ReturnRecovery	DaysRecovery	DaysRecovery
	(1)	(2)	(3)	(4)	(5)	(6)	(7)
LossCarryback	0.0460***	0.3848***	− 117.2785***	0.0264**	0.1334***	− 127.7202***	− 148.8334***
	(4.43)	(4.58)	(− 3.87)	(2.26)	(3.72)	(− 2.85)	(− 2.84)
LossCarryforward	0.0309**	− 0.0210	− 99.5629***	0.0351**	0.1469***	− 193.5319***	− 135.4707**
	(2.54)	(− 0.21)	(− 2.73)	(2.36)	(3.81)	(− 2.75)	(− 1.98)
GroupTaxSystem	0.0065	− 0.1055	− 38.1244	− 0.0134	0.0096	− 90.9661	100.3541*
	(0.65)	(− 1.28)	(− 1.27)	(− 1.20)	(0.29)	(− 1.53)	(1.75)
TaxRate	− 0.5283***	1.5901*	400.6615	− 0.4143***	− 1.1346***	1327.5093**	1966.2293***
	(− 5.18)	(1.92)	(1.35)	(− 3.14)	(− 3.50)	(2.01)	(3.38)
Controls	Yes	Yes	Yes	Yes	Yes	Yes	Yes
Crisis & Industry FE	Yes	Yes	Yes	Yes	Yes	Yes	Yes
Observations	3,139	2,729	2,126	2,717	2,315	1,793	3,139
Centered R^2^	0.1326	0.0765	0.0367	0.1569	0.0887	0.1221	0.0710

This table represents additional robustness checks for Hypothesis 1a and 1b. Columns 1 to 3 (Columns 4 to 6) present the results using no weighting (reduced sample), Column 7 displays results for a modified *DaysRecovery* variable, where outliers and companies which do not reach their pre−  crisis maximum after the respective crisis receive the value of 1,667 days. For the reduced sample, we exclude countries in the top decile of CountryRisk in the year of the crisis. Data from the International Monetary Fund, EY Worldwide Corporate Tax Guide, and Thomson Reuters. The observational units are firms. See Appendix Table 16 for the remaining variable definitions. Heteroskedasticity-robust Driscoll-Kraay standard errors with multi-way clustering on year-industry-level and country-level in parentheses. *Indicates significance at the 10% level, **indicates significance at the 5% level, ***indicates significance at the 1% level

We perform the same set of robustness tests for Hypothesis 2 and 3 and report the respective regression results in Appendix Tables 20 (Hypothesis 2), as well as 21 and 24 (Hypothesis 3). All three robustness tests for Hypothesis 2 support the previous findings of a statistically significant negative coefficient for the interaction term of *TaxRate* and *LossCarryback*, whereas no similar effect is observed for the interaction with *LossCarryforward*. Regarding Hypothesis 3, the estimated coefficient estimates differ in the direction expected for pre-crisis profitability in eight out of twelve specifications. This difference is statistically significant at conventional levels in three out of eight columns. In none of the specifications did we find a statistically significant coefficient estimate pointing in the direction opposite to our expectation.

We perform a second set of robustness tests and report the results in Table 10 below (for Hypothesis 1) and Appendix Tables 23 and 24 in the Appendix (for Hypotheses 2 and 3). In the first three columns of these tables (first six in the case of Appendix Table 24), we include industry-crisis fixed effects in addition to industry and crisis fixed effects. Additionally, controlling for industry-crisis fixed effects follows the notion that industries were affected differently by the two recessions covered by our data. We expect that stock market performance during a recession is significantly influenced by firm risk. While we control for firm beta in the baseline regressions, we incorporate an alternative measure of firm risk (*CompanyRisk*), developed by Hassan et al. (2019), in the remaining specifications of these tables. This proxy captures the share of earnings calls devoted to risk-related topics and hence, measures the overall firm-level risk anticipated by the respective company. Our findings for Hypothesis 1a, 1b, and 2 are generally robust to both of these modifications, even though incorporating *CompanyRisk* reduces the sample size substantially.^20^ As expected, higher-risk companies experience a larger decline and a slower recovery. Our results for these additional robustness tests draw a less clear picture as regards Hypothesis 3, which may well be explained by the smaller sample size. Nonetheless, all three significant differences in the coefficient estimates for *LossCarryback* and *LossCarryforward* point in the expected direction.

In order to illustrate the heterogeneity of effects across crises, we report results for our main specifications testing Hypothesis 1 to 3 also separately for each crisis (see Appendix Tables 25, 26, 27 in the Appendix). These results clearly document that effects differ across crises, as Dobridge (2021) has also observed, and that our previous results do not entirely relate to only one of the two crises. As regards Hypothesis 1, the effects of an unrestricted loss carryback and loss carryforward are considerably stronger during the 2008 crisis than during the 2020 crisis, for which significant effects are estimated only for the dependent variable ReturnRecovery. *GroupTaxSystem* exercises a significant effect only in one out of six specifications. Contrastingly, the results suggest that Hypothesis 2 can be confirmed only for the 2020 crisis, whereas Appendix Table 26 reports results in support of Hypothesis 3 for both crises.

**Table 10:** R﻿obustness tests II Hypothesis 1

Dependent Variable	Additional FEs			CompanyRisk		
	ReturnDecline	ReturnRecovery	DaysRecovery	ReturnDecline	ReturnRecovery	DaysRecovery
	(1)	(2)	(3)	(4)	(5)	(6)
LossCarryback	0.0203	0.2312***	− 189.9162**	0.0281**	0.0691*	− 88.1673*
	(1.63)	(5.02)	(− 2.44)	(2.25)	(1.67)	(− 1.85)
LossCarryforward	0.0231	0.0443	− 161.0860**	0.0276*	0.0734*	− 104.3785*
	(1.56)	(0.78)	(− 2.13)	(1.70)	(1.77)	(− 1.79)
GroupTaxSystem	− 0.0021	0.0266	− 103.9279*	0.0220	− 0.0018	− 56.3473
	(− 0.18)	(0.52)	(− 1.70)	(1.53)	(− 0.05)	(− 1.27)
TaxRate	− 0.5302***	0.0995	888.9939**	− 0.2786*	− 0.7142	757.1467
	(− 4.23)	(0.17)	(2.08)	(− 1.66)	(− 1.56)	(1.47)
CompanyRisk				− 0.0002**	0.0001	0.7986**
				(− 2.09)	(0.37)	(2.34)
Controls	Yes	Yes	Yes	Yes	Yes	Yes
Crisis FE	Yes	Yes	Yes	Yes	Yes	Yes
Industry FE	Yes	Yes	Yes	Yes	Yes	Yes
Crisis-Industry FE	Yes	Yes	Yes	No	No	No
Observations	3139	2729	2126	1056	1029	784
Centered R^2^	0.1387	0.0880	0.1078	0.1823	0.0492	0.0772

This table represents the robustness tests for results reported in Table 3. In Columns 1 to 3, we additionally use crisis-industry fixed effects and Columns 4 to 6 present the results for additionally controlling for *CompanyRisk*. *CompanyRisk* is the firm risk measure developed by Hassan et al. (2019). Data from the International Monetary Fund, EY Worldwide Corporate Tax Guide, and Thomson Reuters. The observational units are firms. See Appendix Table 16 for variable definitions. Heteroskedasticity-robust Driscoll-Kraay standard errors with multi-way clustering on year-industry-level and country-level in parentheses. *Indicates significance at the 10% level, **indicates significance at the 5% level, ***indicates significance at the 1% level.

#### Placebo tests, matching, and synthetic control groups

The cross-sectional structure of our data requires that we comprehensively control for firm-and country-level influences on stock market performance since countries with a generous loss carryback (or loss carryforward) may differ from all other countries in economic terms. In order to further document that our results are not driven by non-tax country differences, we report in this section four additional tests, which address this concern with alternative identification strategies.

**Fig. 2:**
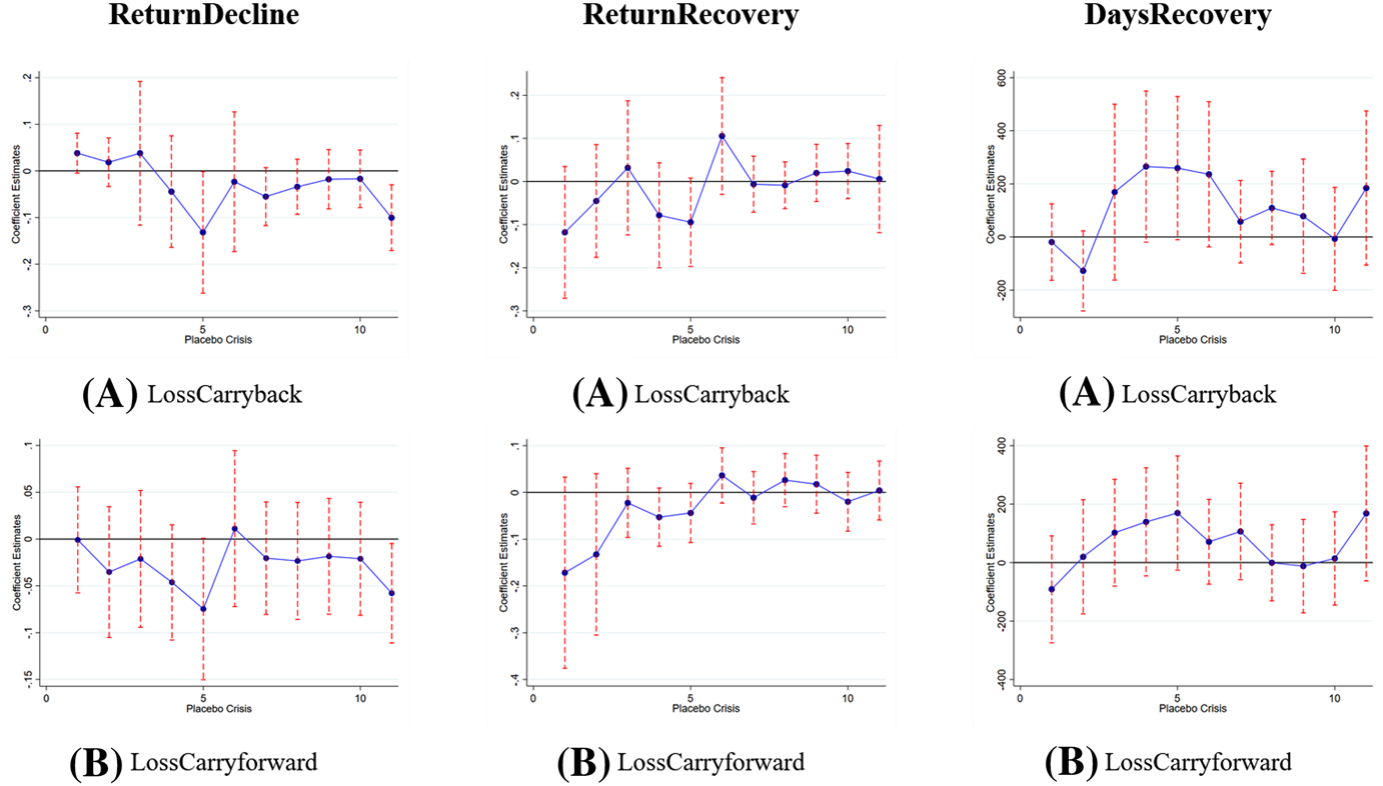
Placebo Tests. Coefficient plots of *LossCarryback* and *LossCarryforward* for all three dependent variables (*ReturnDecline*, *ReturnRecovery*, *DaysRecovery*) for 11 different placebo crises. The dots represent coefficient estimates. Bars depict 95% confidence intervals. Confidence intervals including zero illustrate that we do not find a statistically significant estimate for *LossCarryback* or *LossCarryforward* during our placebo crises

We start with reporting the results of the placebo tests below. To this end, we consider eleven placebo crises of 24 months in length, starting every three months after the end of the 2008 recession.^21^ We run our baseline regression (Eq. 1)^22^ separately for each crisis and report the coefficient estimates and confidence intervals for *LossCarryback* and *LossCarryforward* graphically for all three of our dependent variables. If omitted country influences distorted our results, we would also expect to observe similar effects for at least some of our placebo crises. However, we find that coefficients differ significantly from zero in two of our 66 placebo tests in Fig. 2.

Next, we build synthetic control groups for two countries in our sample (Canada and UK). Both countries allow for an unrestricted loss carryback in both crises and have the largest benchmark indices resulting in the highest number of country-specific observations. We consider all countries without unrestricted loss carryback as potential members for the synthetic control group, which we build based on all country control variables, including loss carryforward and group tax system. We run separate regressions for our two case study countries and their respective synthetic control group and report the results in Table 11 below. For Canada (the UK), five (four) out of six coefficient estimates of *LossCarryback* and *LossCarryforward* point in the expected direction. These findings further support the validity of our baseline results, particularly since the sample size for these case studies is considerably smaller. However, only three out of nine of these coefficient estimates are also statistically significant at conventional levels.

**Table 11:** Synthetic control-  case studies

	Canada			UK		
	ReturnDecline	ReturnRecovery	DaysRecovery	ReturnDecline	ReturnRecovery	DaysRecovery
	(1)	(2)	(3)	(4)	(5)	(6)
LossCarryback	0.1849***	0.1385	411.7789**	0.0162	− 0.0579	98.8985
	(3.10)	(0.60)	(2.03)	(0.70)	(− 0.66)	(1.11)
LossCarryforward	0.0027	0.3987	− 172.5136	0.0849***	0.0408	− 150.7022*
	(0.05)	(1.63)	(− 1.45)	(3.20)	(0.45)	(− 1.74)
GroupTaxSystem	0.1209**	− 0.1896	174.9703	− 0.0762***	− 0.0961	− 29.3059
	(2.47)	(− 0.83)	(1.32)	(− 2.81)	(− 1.21)	(− 0.35)
Firm Controls	Yes	Yes	Yes	Yes	Yes	Yes
Crisis & Industry FE	Yes	Yes	Yes	Yes	Yes	Yes
Observations	460	441	319	367	364	282
R^2^	0.2051	0.1323	0.1176	0.1202	0.0497	0.0441

This table represents synthetic control case studies for Canada and the UK. Data from the International Monetary Fund, EY Worldwide Corporate Tax Guide, and Thomson Reuters. The observational units are firms. See Appendix Table 16 for variable definitions. Heteroskedasticity-robust Driscoll-Kraay standard errors with multi-way clustering on year-industry-level and country-level in parentheses. *Indicates significance at the 10% level, **indicates significance at the 5% level, ***indicates significance at the 1% level

In Table 12, we use a matched sample of firms that were always profitable during the period under consideration and firms that experienced a one-time loss during the respective crisis. We match companies based on ReturnPreCrisis within the same industry to ensure companies with a comparable capital market performance of the same industry. This robustness test follows the idea that these two subsets of firms are generally comparable in terms of their overall profitability despite being affected differently by loss offset regulations. Although firms with a one-time loss during the crisis benefit only to some extent from a generous loss offset and the sample size is small, all six coefficient estimates for this sub-sample of firms point in the expected direction; two of these estimates are also significant at conventional levels. We find no similar effects for the matched sample of firms without any losses.

**Table 12:** One-time losses vs. always profitable companies

Dependent Vvariable	ReturnDecline		ReturnRecovery		DaysRecovery	
	OneTimeLoss	AlwaysProfits	OneTimeLoss	AlwaysProfits	OneTimeLoss	AlwaysProfits
LossCarryback	0.0209	− 0.0235	0.2669*	0.1617	− 62.9963	− 62.0762
	(0.71)	(− 0.70)	(1.76)	(1.22)	(− 0.42)	(− 0.73)
LossCarryforward	0.0703**	− 0.0880**	0.1179	− 0.2843*	− 57.6332	109.3245
	(2.08)	(− 2.12)	(0.68)	(− 1.71)	(− 0.34)	(1.02)
GroupTaxSystem	− 0.0017	− 0.0220	0.0700	− 0.2928**	− 40.0932	85.2200
	(− 0.06)	(− 0.69)	(0.50)	(− 2.26)	(− 0.29)	(1.02)
TaxRate	− 0.6728**	− 0.1567	0.4001	0.7205	− 589.2382	581.8088
	(− 2.15)	(− 0.48)	(0.25)	(0.55)	(− 0.39)	(0.64)
*F−Test*						
LCB=LCF	0.1455	0.1006	0.3935	0.0048***	0.9737	0.0958*
Controls	Yes	Yes	Yes	Yes	Yes	Yes
Crisis & Industry FE	Yes	Yes	Yes	Yes	Yes	Yes
Observations	315	315	253	281	136	219
Centered R^2^	0.1552	0.2362	0.1464	0.1427	0.1249	0.1671

This table represents additional results for Hypothesis 3. The sample consists of companies experiencing a one−  time loss in the crisis year and matched with always profitable firms. Data from the International Monetary Fund, EY Worldwide Corporate Tax Guide, and Thomson Reuters. The observational units are firms. See Appendix Table 16 for variable definitions. Heteroskedasticity-robust Driscoll-Kraay standard errors with multi-way clustering on year-industry-level and country-level in parentheses. *Indicates significance at the 10% level, **indicates significance at the 5% level, ***indicates significance at the 1% level

Finally, we exploit within-country heterogeneity in Table 13 to further document that our findings for Hypothesis 1a are not driven by economic differences across countries. We change our regression design and split the sample based on our main explanatory variables, the availability of an unrestricted loss carryback (*LossCarryback*, upper section of Table 13) or unrestricted loss carryforward (*LossCarryforward*, lower section of Table 13). Our dependent variables are still our three performance measures, *ReturnDecline*, *ReturnRecovery*, and *DaysRecovery*. Our main independent variable in this Table is *HighProfits*, an indicator variable that equals one if a firm belongs to the 50% of firms with the highest pre-crisis profitability. If generous loss carryover regulations effectively mitigate the negative effects of having accounting losses, then the stock price development should depend to a smaller extent on firm profitability if countries offer an unrestricted carryback or carryforward of tax losses. Our results support this theoretical prediction. Whereas in countries without unrestricted loss carryback or unrestricted loss carryforward, the stock price development during the crisis is significantly better for the high-profitability subset of firms, *HighProfits* is statistically significant in only two out of six specifications referring to countries with unrestricted loss carryover rules.

**Table 13:** Profitability bins

LossCarryback						
Dependent Variable	ReturnDecline		ReturnRecovery		DaysRecovery	
	LossCarryback	No LossCarryback	LossCarryback	No LossCarryback	LossCarryback	No LossCarryback
HighProfits	0.0558***	0.0558***	0.0437	0.1659***	− 50.7374	− 142.2819***
	(2.91)	(5.24)	(0.92)	(3.34)	(− 1.15)	(− 2.81)
Controls	Yes	Yes	Yes	Yes	Yes	Yes
Crisis & Industry FE	Yes	Yes	Yes	Yes	Yes	Yes
Observations	566	2,572	559	2,169	426	1,699
Centered R^2^	0.1389	0.1843	0.0629	0.1067	0.0492	0.1415

LossCarryforward						
Dependent Variable	ReturnDecline		ReturnRecovery		DaysRecovery	
	LossCarryforward	No LossCarryforward	LossCarryforward	No LossCarryforward	LossCarryforward	No LossCarryforward
HighProfits	0.0486***	0.0599***	− 0.0017	0.1718***	− 49.8320	− 135.7586**
	(3.00)	(5.80)	(− 0.03)	(3.37)	(− 0.86)	(− 2.18)
Controls	Yes	Yes	Yes	Yes	Yes	Yes
Crisis & Industry FE	Yes	Yes	Yes	Yes	Yes	Yes
Observations	540	2,599	524	2,205	353	1,773
Centered R^2^	0.2209	0.1994	0.1491	0.1289	0.1231	0.1241

This table represents the results of an additional profitability analysis, including all sample countries. *HighProfits* is an indicator variable taking the value of 1 if the respective company’s profit is above the median, with profit defined as net income scaled by total assets where both values correspond to the pre-crisis. Observations are allocated into the low and high categories based on a median split. Data from the International Monetary Fund, EY Worldwide Corporate Tax Guide, and Thomson Reuters. The observational units are firms. See Appendix Table 16 for variable definitions. Heteroskedasticity-robust Driscoll-Kraay standard errors with multi-way clustering on year-industry-level and country-level in parentheses. *Indicates significance at the 10% level, **indicates significance at the 5% level, ***indicates significance at the 1% level.

## Conclusion

This paper presents one of the first empirical analyzes of the stabilizing effect of more generous loss offset regulations on firm development during two recent economic crises. We analyze the stock market development of 2729 listed firms from 24 industrialized countries to test three hypotheses. First, we expect that both an unrestricted loss carryback and an unrestricted loss carryforward are associated with a weaker stock price decline during the crises, as well as a stronger and more timely recovery post-crisis. Second, we expect this effect to be more pronounced in high-tax countries. Third, we assume that a loss carryback is relevant rather for firms with pre-crisis profits, whereas firms with pre-crisis losses benefit more from an unrestricted loss carryforward. Our empirical analysis supports all three hypotheses with statistical significance at conventional levels. Our estimation results underline that effects are also of economically relevant size. Firms that benefit from an unrestricted loss carryforward or unrestricted loss carryback show a stock price decline during the crisis by more than two percentage points lower. Besides, the recovery period is more than 160 days shorter. Our results thus provide clear empirical evidence of the stabilizing effects of a generous loss offset.

Besides the stabilizing effects of a generous loss offset for firm development during crises, our results also clearly document that investors consider the (complex) effects of intertemporal loss offset. This is an important finding with policy implications since a stable stock market development may help stabilize consumption (and thus the overall development of the economy) during an economic crisis.

We do not withhold the potential limitations of our study, resulting mainly from the structure of our data. Using a cross-section model has apparent weaknesses in identifying causal effects. In particular, we cannot control for the scope of other policy measures to recover the economy during a crisis, which may well correlate with the generosity of tax loss offset rules. We address this concern, as well as the general concern that our regressions capture unobserved country characteristics, by presenting a comprehensive set of robustness tests for all three hypotheses, including modifications of the sample, regressions exploiting within-country heterogeneity as well as regressions based on a matched sample or using a synthetic control group. The big picture of all results presented in this paper confirms that the observed effects cannot be traced to such influences.

## Data Availability

The data that support the findings of this study are available from Thomson Reuters but restrictions apply to the availability of these data, which were used under license for the current study, and so are not publicly available. Data from the EY Worldwide Corporate Tax Guides, KPMG, and the IMF are available from the corresponding author on reasonable request.

## Appendix

See below Appendix Tables 14, 15, 16, 17, 18, 19, 20, 21, 22, 23, 24, 25, 26 and 27.

**Table 14:** Tax rate and loss carryover rules country overview

Country	TaxRate		LossCarryback				LossCarryforward			
	2007	2019	2007		2019		2007		2019	
			Limit	Years	Limit	Years	Limit	Years	Limit	Years
Australia	30	30	n.a.	0	n.a.	0	0	$\infty$	0	$\infty$
Belgium	33	29	n.a.	0	n.a.	0	0	$\infty$	0	$\infty$
Brasil	15	34	n.a.	0	n.a.	0	1	$\infty$	1	$\infty$
Canada	22.12	26.5	0	3	0	3	0	20	0	20
China	30	25	n.a.	0	n.a.	0	0	5	0	5
Czech Republic	24	19	n.a.	0	n.a.	0	0	5	0	5
France	33.3	31	1	3	1	1	0	$\infty$	1	$\infty$
Germany	25	30	1	1	1	1	1	$\infty$	1	$\infty$
Greece	25	24	n.a.	0	n.a.	0	0	5	0	5
India	30	30	n.a.	0	n.a.	0	0	8	0	8
Indonesia	30	25	n.a.	0	n.a.	0	0	5	0	5
Japan	30	30.62	0	1	0	1	0	7	1	10
Mexico	28	30	n.a.	0	n.a.	0	0	10	0	10
Netherlands	25.5	25	0	1	0	1	0	9	0	9
Poland	19	19	n.a.	0	n.a.	0	0	5	1	5
Portugal	25	21	n.a.	0	n.a.	0	0	6	1	5
Russia	20	20	n.a.	0	n.a.	0	0	10	1	$\infty$
Saudi Arabia	20	20	n.a.	0	n.a.	0	1	$\infty$	1	$\infty$
South Africa	29	28	n.a.	0	n.a.	0	0	$\infty$	0	$\infty$
Spain	32.5	25	n.a.	0	n.a.	0	0	15	1	$\infty$
Sweden	28	21.4	n.a.	0	n.a.	0	0	$\infty$	0	$\infty$
Turkey	20	22	n.a.	0	n.a.	0	0	5	0	5
United Kingdom	30	19	0	1	0	1	0	$\infty$	1	$\infty$
USA	35	21	0	2	n.a.	0	0	20	1	$\infty$

This table displays the sample countries with detailed information on *TaxRate*, *LossCarryback*, and *LossCarryforward*. *TaxRate* is the country’s statutory tax rate in the respective year. *Limit* is a dummy variable that equals one if the *LossCarryback* or *LossCarryforward* is amount restricted. *Years* is the number of years a *LossCarryback* or *LossCarryforward *can be used. *n.a.* represents no value for this item and $\infty$

represents unrestricted time use of *LossCarryforwards.*

**Table 15:** Country overview

Country	Index	LossCarryback		LossCarryforward		GroupTaxSystem		IncreasedLossOffset	
		2007	2019	2007	2019	2007	2019	2008	2020
Australia	ASX All Ordinaries	0	0	1	1	1	1	0	1
Belgium	BEL 20	0	0	1	1	0	0	0	0
Brasil	Bovespa Index	0	0	0	0	0	0	0	0
Canada	S &P TSX Composite	1	1	0	0	0	0	0	0
China	SSE Composite Index	0	0	0	0	0	0	0	1
Czech Republic	Czech Traded Index	0	0	0	0	0	0	0	1
France	CAC 40	0	0	1	0	1	1	1	0
Germany	DAX 30	0	0	0	0	1	1	0	0
Greece	Athex 20	0	0	0	0	0	0	0	0
India	Nifty	0	0	0	0	0	0	0	0
Indonesia	IDX Composite	0	0	0	0	0	0	0	0
Japan	NIKKEI 225	1	1	0	0	1	1	1	0
Mexico	S &P BMV IPC	0	0	0	0	1	1	0	0
Netherlands	AEX	1	1	0	0	1	1	0	1
Poland	WIG20	0	0	0	0	1	1	0	0
Portugal	PSI 20	0	0	0	0	1	1	0	1
Russia	MOEX	0	0	0	0	0	0	0	0
Saudi Arabia	Tadawul All-Share Index	0	0	0	0	0	0	0	0
South Africa	Johannesburg Stock Exchange	0	0	1	1	1	1	0	0
Spain	IBEX 35	0	0	0	0	1	1	0	0
Sweden	OMX Sockholm 30	0	0	1	1	1	1	0	0
Turkey	BIST 100	0	0	0	0	0	0	0	0
United Kingdom	FTSE 100	1	1	1	0	1	1	0	0
USA	Dow Jones Industrial Average	1	0	0	0	0	0	0	1

This table displays the sample countries with their benchmark index names. *LossCarryback* is a dummy variable that equals one if the firm’s home country offers a loss carryback of at least one year that is not restricted in amount and zero otherwise. *LossCarryforward* takes the value of one if the firm’s home country allows for a loss carryforward that is restricted neither in terms of time nor amount and zero otherwise. *GroupTaxSystem *is an indicator variable taking the value of one if the firm’s home country provides a group tax system regulation and zero otherwise. *IncreasedLossOffset* is a dummy variable that equals one if the respective country increased its LossCarryback or LossCarryforward regulations during the course of the crisis. We only consider changes with unrestricted amounts

**Table 16:** Descriptive statistics

Variable	Obs.	Mean	SD	min	p50	max
ReturnDecline	3139	− 0.49	0.20	− 1.00	− 0.46	− 0.00
ReturnRecovery	2729	1.11	1.31	0.00	0.90	24.94
DaysRecovery	2126	355.13	465.71	1.00	210.00	3,273.00
LossCarryback	3139	0.18	0.38	0.00	0.00	1.00
LossCarryforward	3139	0.17	0.38	0.00	0.00	1.00
Combined1	3139	2.35	0.54	1	2	4
Combined2	3139	2.33	0.61	1	2	3.8
GroupTaxSystem	3139	0.27	0.45	0.00	0.00	1.00
TaxRate	3139	0.26	0.04	0.15	0.25	0.35
Unemployment	3139	6.85	5.01	2.01	5.15	28.47
GDPperCapita	3139	4.60	3.35	0..74	3.14	10.89
Inflation	3139	3.13	2.50	− 2.09	2.90	15.18
GDPGrowth	3139	4.08	2.11	− 0.05	5.02	7.66
ExchangeRate	3139	1236.57	3652.62	0.50	6.91	14147.67
CountryRisk	3139	16.23	3.94	7.00	17.00	21.00
Population	3139	570.50	629.33	9.22	192.03	1402.11
Beta	3139	0.98	0.51	− 2.34	0.98	4.46
Leverage	3139	0.27	1.12	0.00	0.23	61.66
Size	3139	16.67	2.65	8.98	16.14	26.11
ReturnPreCrisis	3139	0.81	2.23	0.00	0.36	54.11
ChangeUnemployment	3139	0.08	0.30	− 0.23	0.07	1.17
ChangeGDPperCapita	3139	0.11	0.12	− 0.22	0.12	0.33
CompanyRisk	1056	83.64	46.59	0	75.52	459.77

Descriptive statistics of main dependent and explanatory variables. Macroeconomic country variables from the International Monetary Fund, data on loss carryovers and group taxation from the EY Worldwide Corporate Tax Guide. Data on the corporate tax rates by KPMG and stock market and firm data from Thomson Reuters. Own calculations. *ReturnDecline* is the percentage share price decline during the crisis period, calculated as the difference between the minimum stock price during the crisis and the maximum stock price reached prior to the crisis (adjusted for dividend distributions). *ReturnRecovery* is measured as the percentage difference between the pre-crisis maximum and the stock price at the number of days the MSCI World needed to recover from the respective crisis after the crisis minimum. *DaysRecovery* is the number of days between the day of the minimum stock price during the respective crisis period and the day at which the stock price reaches its pre-crisis maximum. *LossCarryback* is a dummy variable that equals one if the firm’s home country in the year prior to the crisis offers a loss carryback of at least one year that is not restricted in amount and zero otherwise. *LossCarryforward* takes the value of 1 if the firm’s home country in the year prior to the crisis allows for a loss carryforward that is restricted neither in terms of time nor amount and zero otherwise. *Combined1* is a score-based measure with values of 0, 1, 2, or 4. The score increases by one point for a loss carryback of at least one year as well as a loss carryforward without time restriction. One further point is added for each of these regulations not being restricted in amount *Combined2* further differentiates depending on time-related restrictions. Whereas again, one point is granted for loss carryback or loss carryforward without amount-related restriction, values between zero and one are added to the score depending on the loss carryforward and loss carryback periods. *GroupTaxSystem* is an indicator variable taking the value of one if the firm’s homecountry provides a group tax system regulation and zero otherwise. *TaxRate* is the statutory tax rate of the home country in the respective crisis. Unemployment, *GDPperCapita*, and Inflation represent the home country’s unemployment rate, gross domestic product per capita, and the inflation rate for the crisis, respectively. *GDPGrowth* is the home country’s change in GPD, *ExchangeRate* is the exchange rate of the national currency in terms of US dollar, *CountryRisk* is the home country’s Moody’s rating, and *Population* is the home country’s number of citizens in millions. *Beta, Leverage*, and *Size* are the firm’s beta coefficient, total debt to total assets ratio, and total assets, respectively. All country and firm control variables are used as lagged variables. *ReturnPreCrisis* is the firm’s stock market return prior to the respective crisis. *ChangeUnemployment* is the change in the country’s unemployment rate between one year prior to and after the crisis. *ChangeGDPperCapita* is the change in the country’s GDP per capita between one year prior to and after the crisis. *CompanyRisk* is the firm risk measure developed by Hassan et al. (2019). It captures the company’s anticipated overall firm-level risk

**Table 17:** Correlation matrix

	(1)	(2)	(3)	(4)	(5)	(6)	(7)	(8)	(9)	(10)
(1) LossCarryback	1.000									
(2) LossCarryforward	**− 0.058**	1.000								
(3) GroupTaxSystem	**0.326**	**0.494**	1.000							
(4) TaxRate	0.032	**0.391**	**0.327**	1.000						
(5) Unemployment	**− 0.219**	**0.368**	**0.212**	**0.075**	1.000					
(6) GDPperCapita	**0.267**	**0.437**	**0.451**	**0.136**	**0.179**	1.000				
(7) Inflation	**− 0.268**	**− 0.041**	**− 0.178**	**− 0.043**	**0.398**	**0.054**	1.000			
(8) GDPGrowth	**− 0.345**	**− 0.218**	**− 0.483**	**− 0.200**	**− 0.073**	0.031	**0.190**	1.000		
(9) ExchangeRate	**− 0.155**	**− 0.154**	**− 0.205**	**0.072**	**− 0.097**	**− 0.076**	**0.167**	**0.231**	1.000	
(10) CountryRisk	**− 0.396**	0.013	**− 0.212**	**0.037**	**0.438**	0.438	**0.797**	**0.169**	**0.424**	1.000
(11) Population	**− 0.370**	**− 0.387**	**− 0.491**	**− 0.089**	**− 0.271**	**− 0.586**	− 0.006	**0.596**	**− 0.166**	**− 0.162**
(12) Beta	**0.046**	− 0.010	− 0.017	0.030	**− 0.137**	0.007	**− 0.080**	**0.040**	**− 0.226**	**− 0.163**
(13) Leverage	− 0.001	− 0.013	− 0.006	− 0.007	− 0.014	− 0.005	0.004	− 0.008	**0.073**	0.021
(14) Size	**0.074**	**− 0.180**	**0.095**	**0.254**	**− 0.165**	**− 0.142**	**0.043**	**− 0.080**	**0.599**	**0.223**
(15) ReturnPreCrisis	**− 0.045**	**0.131**	**0.092**	**0.045**	**0.143**	**0.274**	**0.116**	**0.068**	**0.044**	**0.180**
(16) ChangeUnemployment	**0.375**	**0.105**	**0.286**	**0.108**	**− 0.129**	**0.197**	**− 0.098**	**− 0.322**	**0.423**	**0.072**
(17) ChangeGDPperCapita	**− 0.444**	**− 0.183**	**− 0.408**	**− 0.078**	**− 0.057**	**− 0.433**	**0.108**	**0.504**	**0.151**	**− 0.143**

	(11)	(12)	(13)	(14)	(15)	(16)	(17)			
(1) LossCarryback										
(2) LossCarryforward										
(3) GroupTaxSystem										
(4) TaxRate										
(5) Unemployment										
(6) GDPperCapita										
(7) Inflation										
(8) GDPGrowth										
(9) ExchangeRate										
(10) CountryRisk										
(11) Population	1.000									
(12) Beta	**0.156**	1.000								
(13) Leverage	− 0.035	− 0.020	1.000							
(14) Size	**− 0.123**	**− 0.107**	0.017	1.000						
(15) ReturnPreCrisis	**− 0.159**	**0.064**	− 0.010	**− 0.077**	1.000					
(16) ChangeUnemployment	**− 0.609**	**− 0.158**	**0.057**	**0.324**	**0.049**	1.00				
(17) ChangeGDPperCapita	**0.548**	**0.041**	− 0.017	0.001	− 0.023	**− 0.620**	1.000			

This table provides pairwise correlations for the sample

Bold values represent statistical significance at 5% level

**Table 18:** Detailed descriptive statistics

Variable	LossCarryback = 1			LossCarryback = 0			Diff
	Mean	SD	p50	Mean	SD	p50	
ReturnDecline	− 0.50	0.19	− 0.46	− 0.48	0.20	− 0.46	− 0.02
ReturnRecovery	1.02	0.62	0.90	1.13	1.44	0.90	− 0.11
DaysRecovery	450.26	494.99	303	331.22	455.09	189	− 119.04**
LossCarryback	1	0	1	0	0	0	1
LossCarryforward	0.13	0.33	0	0.18	0.39	0	− 0.05**
GroupTaxSystem	0.58	0.49	1	0.21	0.40	0	0.37**
TaxRate	0.26	0.04	0.27	0.26	0.04	0.25	0.00
Unemployment	4.51	1.42	5.3	7.36	5.36	5.15	− 2.85**
GDPperCapita	6.51	3.42	3.84	4.18	3.19	2.32	− 2.33**
Inflation	1.70	0.75	1.95	3.44	2.64	2.90	− 1.74**
GDPGrowth	2.53	2.30	1.86	4.42	1.90	5.36	− 1.89**
ExchangeRate	27.47	46.51	1.07	1503.11	3986.23	6.90	− 1457.64**
CountryRisk	19.68	1.79	21.00	15.47	3.88	17.00	4.21**
Population	73.99	59.58	61.81	679.96	645.20	273.52	− 605.97**
Beta	1.03	0.53	0.98	0.97	0.50	0.98	0.06**
Leverage	0.26	0.17	0.25	0.26	1.23	0.22	0.00
Size	17.08	2.75	16.27	16.57	2.62	16.11	0.51**
ReturnPreCrisis	0.59	1.25	0.36	0.85	2.39	0.36	− 0.26**
CompanyRisk	87.61	50.33	79.23	80.89	43.66	74.52	6.72**
ChangeUnemployment	0.32	0.17	0.31	0.03	0.30	− 0.03	0.29**
ChangeGDPperCapita	− 0.01	0.11	− 0.02	0.13	0.11	0.15	− 0.14**
Liquidity	0.11	0.12	0.08	0.10	0.13	0.06	0.01

Variable	LossCarryforward = 1			LossCarryforward = 0			Diff
	Mean	SD	p50	Mean	SD	p50	
ReturnDecline	− 0.56	0.21	− 0.56	− 0.47	0.19	− 0.45	− 0.09**
ReturnRecovery	1.05	0.82	0.91	1.12	1.41	0.90	− 0.07
DaysRecovery	547.84	626.21	367	311.38	413.12	190	236.46**
LossCarryback	0.13	0.34	0	0.19	0.39	0	0.06**
LossCarryforward	1	0	1	0	0	0	1
GroupTaxSystem	0.76	0.43	1	0.17	0.38	0	0.59**
TaxRate	0.29	0.02	0.30	0.26	0.04	0.25	0.03**
Unemployment	10.89	9.49	5.30	6.01	2.75	5.15	4.88**
GDPperCapita	7.81	3.29	8.72	3.93	2.96	2.32	3.88**
Inflation	2.90	1.68	2.30	3.17	2.64	2.90	− 0.27**
GDPGrowth	3.07	1.44	2.42	4.29	2.16	5.95	− 1.22**
ExchangeRate	1.44	4.11	3.73	1492.72	3966.51	6.91	− 1491.28**
CountryRisk	19.22	3.21	21.00	15.61	3.79	17.00	3.61**
Population	36.47	19.00	25.69	681.46	637.75	273.52	− 644.99**
Beta	0.97	0.57	0.89	0.99	0.50	0.99	− 0.02
Leverage	0.23	0.19	0.22	0.27	1.22	0.23	− 0.04
Size	15.62	1.77	15.58	16.88	2.75	16.31	− 1.26**
ReturnPreCrisis	1.45	3.68	0.65	0.67	1.76	0.31	0.78**
CompanyRisk	83.22	45.16	76.44	83.81	47.20	75.27	− 0.59
ChangeUnemployment	0.15	0.19	0.20	0.07	0.32	− 0.03	0.08**
ChangeGDPperCapita	0.06	0.13	0.12	0.12	0.12	0.12	− 0.06**
Liquidity	0.10	0.12	0.07	0.10	0.13	0.02	0.00

Descriptive statistics of main dependent and explanatory variables by group. See Appendix Table 16 for variable definitions. *Liquidity* is the post-crisis level of cash holdings scaled by total assets. **labels statistical significance at 5% level

**Table 19:** Heterogeneity in loss offset possibilities II

Dependent Variable	ReturnDecline	ReturnRecovery	DaysRecovery
	(1)	(2)	(3)
Combined2	0.0258***	0.1601***	− 126.7536**
	(3.37)	(5.35)	(− 2.56)
GroupTaxSystem	0.0103	0.1150**	− 170.8648**
	(0.85)	(2.38)	(− 2.35)
TaxRate	− 0.5768***	− 0.3988	1154.0809**
	(− 4.50)	(− 0.76)	(2.36)
Controls	Yes	Yes	Yes
Crisis & Industry FE	Yes	Yes	Yes
Observations	3,139	2,729	2,126
Centered R^2^	0.1395	0.0786	0.1052

This table represents the heterogeneity analysis for *LossCarryback* and *LossCarryforward* time and amount restrictions. Data from the International Monetary Fund, EY Worldwide Corporate Tax Guide, and Thomson Reuters. The observational units are firms. *Combined2* is a score-based measure with values out of the real interval from zero to four, including the boundaries. The score increases by one point for unrestricted loss carryback and loss carryforward regulations regarding the amount. Additionally, values between zero and one are added to the score depending on the loss carryforward periods (5 years: 0, 6 years: 0.05, 8 years: 0.15, 9 years: 0.2, 10 years: 0.25, 15 years: 0.5, 20 years: 0.8, unlimited: 1) and loss carryback periods (1 year: 0.1, 2 years: 0.5, 3 years: 1). Since this variable is not restricted to integer values, we include it as a metric variable. See Appendix Table 16 for the remaining variable definitions. Heteroskedasticity-robust Driscoll-Kraay standard errors with multi-way clustering on year-industry-level and country-level in parentheses. *Indicates significance at the 10% level, **indicates significance at the 5% level, ***indicates significance at the 1% level

**Table 20:** Robustness tests I Hypothesis 2

Dependent Variable	Unweighted			Reduced Sample			Full Sample
	ReturnDecline	ReturnRecovery	DaysRecovery	ReturnDecline	ReturnRecovery	DaysRecovery	DaysRecovery
	(1)	(2)	(3)	(4)	(5)	(6)	(7)
LossCarryback#TaxRate	0.7134***	0.1615	− 1889.7243***	0.4726*	0.9379	− 1569.5193*	− 3314.5849**
	(3.54)	(0.10)	(− 3.17)	(1.90)	(1.08)	(− 1.91)	(− 2.97)
LossCarryforward#TaxRate	− 0.3119	− 9.5221***	2481.0920**	− 0.4423	0.1381	− 1887.8206	2662.3760*
	(− 0.84)	(− 3.15)	(2.43)	(− 1.10)	(0.12)	(− 0.95)	(1.76)
LossCarryback	− 0.1413***	0.3422	381.6765**	− 0.0946	− 0.1102	284.2698	699.0406**
	(− 2.62)	(0.78)	(2.39)	(− 1.44)	(− 0.49)	(1.39)	(2.31)
LossCarryforward	0.1196	2.7608***	− 812.3329***	0.1582	0.1088	330.5344	− 893.4922**
	(1.10)	(3.10)	(− 2.73)	(1.42)	(0.36)	(0.66)	(− 2.04)
GroupTaxSystem	0.0088	− 0.1504*	− 32.5229	− 0.0157	0.0163	− 124.7991	109.7565*
	(0.85)	(− 1.78)	(− 1.05)	(− 1.22)	(0.41)	(− 1.54)	(1.83)
TaxRate	− 0.6246***	2.2722**	469.2807	− 0.4235**	− 1.3788**	2145.0671*	2089.8874***
	(− 5.57)	(2.49)	(1.44)	(− 1.22)	(0.41)	(− 1.54)	(3.00)
Controls	Yes	Yes	Yes	Yes	Yes	Yes	Yes
Crisis & Industry FE	Yes	Yes	Yes	Yes	Yes	Yes	Yes
Observations	3139	2729	2126	2717	2315	1793	3,139
Centered R^2^	0.1367	0.0818	0.0449	0.1619	0.0898	0.1280	0.0826

This table represents additional robustness checks for Hypothesis 2. Columns 1 to 3 (Columns 4 to 6) present the results using no weighting (reduced sample), Column 7 displays results for a modified *DaysRecovery* variable, where outliers and companies which do not reach their pre-crisis maximum after the respective crisis receive the value of 1,667 days. For the reduced sample, we exclude countries in the top decile of CountryRisk in the year of the crisis. Data from the International Monetary Fund, EY Worldwide Corporate Tax Guide, and Thomson Reuters. The observational units are firms. See Appendix Table 16 for the remaining variable definitions. Heteroskedasticity-robust Driscoll-Kraay standard errors with multi-way clustering on year-industry-level and country-level in parentheses. *Indicates significance at the 10% level, **indicates significance at the 5% level, ***indicates significance at the 1% level

**Table 21:** Robustness tests Ia Hypothesis 3

Dependent Variable	Unweighted						Full Sample	
	ReturnDecline	ReturnDecline	ReturnRecovery	ReturnRecovery	DaysRecovery	DaysRecovery	DaysRecovery	DaysRecovery
	Loss	Profit	Loss	Profit	Loss	Profit	Loss	Profit
LossCarryback	0.1322**	0.0388***	0.8777	0.3542***	142.7991	− 123.6221***	− 184.0016	− 145.7299***
	(2.16)	(3.74)	(1.39)	(4.27)	(0.64)	(− 4.04)	(− 0.87)	(− 2.78)
LossCarryforward	0.1466**	0.0215*	0.7985	− 0.0559	7.9542	− 102.7876***	− 792.1996***	− 107.6790
	(2.00)	(1.77)	(1.02)	(− 0.57)	(0.03)	(− 2.80)	(− 3.68)	(− 1.52)
GroupTaxSystem	0.1013*	0.0025	− 0.2432	− 0.1114	− 385.5632*	− 42.4271	70.1343	109.9688*
	(1.79)	(0.25)	(− 0.41)	(− 1.36)	(− 1.77)	(− 1.39)	(0.36)	(1.88)
TaxRate	− 1.4765***	− 0.4650***	− 0.9191	1.5854*	891.0601	505.1686*	3199.8805	2005.6991***
	(− 2.88)	(− 4.52)	(− 0.17)	(1.91)	(0.52)	(1.66)	(1.64)	(3.38)
*F−Test*								
LCB=LCF	0.8084	0.1625	0.9021	0.0000***	0.4774	0.5667	0.0197**	0.5836
Controls	Yes	Yes	Yes	Yes	Yes	Yes	Yes	Yes
Crisis & Industry FE	Yes	Yes	Yes	Yes	Yes	Yes	Yes	Yes
Observations	253	2,882	212	2,513	147	1,976	264	2,885
Centered R^2^	0.1787	0.1316	0.0724	0.0827	0.1393	0.0438	0.1911	0.0696

This table represents additional robustness checks for Hypothesis 3. Columns 1 to 6 present the results using no weighting, Columns 7 and 8 display results for a modified *DaysRecovery*variable, where outliers and companies which do not reach their pre−  crisis maximum after the respective crisis received the value of 1,667 days. Data from the International Monetary Fund, EY Worldwide Corporate Tax Guide, and Thomson Reuters. The observational units are firms. See Appendix Table 16 for the remaining variable definitions. Heteroskedasticity-robust Driscoll-Kraay standard errors with multi-way clustering on year-industry-level and country-level in parentheses. *Indicates significance at the 10% level, **indicates significance at the 5% level, ***indicates significance at the 1% level

**Table 22:** Robustness tests Ib Hypothesis 3

Dependent Variable	Reduced Sample					
	ReturnDecline	ReturnDecline	ReturnRecovery	ReturnRecovery	DaysRecovery	DaysRecovery
	Loss	Profit	Loss	Profit	Loss	Profit
LossCarryback	0.1933***	0.0203*	0.5002***	0.1142***	− 489.7707	− 122.2210***
	(3.31)	(1.77)	(2.89)	(3.10)	(− 0.69)	(− 2.77)
LossCarryforward	0.2377***	0.0259*	0.8042***	0.1227***	− 399.4185	− 189.5602***
	(3.27)	(1.65)	(3.51)	(3.04)	(− 0.72)	(− 2.66)
GroupTaxSystem	0.0828	− 0.0156	0.1251	0.0203	− 597.8438	− 97.1340
	(1.40)	(− 1.42)	(0.64)	(0.60)	(− 0.76)	(− 1.58)
TaxRate	− 0.1287	− 0.4232***	1.1477	− 1.2654***	− 886.4008	1440.4310**
	(− 0.17)	(− 3.29)	(0.52)	(− 3.83)	(− 0.30)	(2.17)
*F−Test*						
LCB=LCF	0.4694	0.7428	0.0990*	0.8485	0.8300	0.1596
Controls	Yes	Yes	Yes	Yes	Yes	Yes
Crisis & Industry FE	Yes	Yes	Yes	Yes	Yes	Yes
Observations	201	2,513	161	2,151	111	1,680
Centered R^2^	0.4393	0.1603	0.4182	0.1023	0.5190	0.1255

This table represents additional robustness checks for Hypothesis 3 using a reduced sample excluding countries in the top decile of CountryRisk in the year of the crisis. Data from the International Monetary Fund, EY Worldwide Corporate Tax Guide, and Thomson Reuters. The observational units are firms. See Appendix Table 16 for the remaining variable definitions. Heteroskedasticity-robust Driscoll-Kraay standard errors with multi-way clustering on year-industry-level and country-level in parentheses. *Indicates significance at the 10% level, **indicates significance at the 5% level, ***indicates significance at the 1% level

**Table 23:** Robustness tests II Hypothesis 2

Dependent Variable	Additional FEs			CompanyRisk		
	ReturnDecline	ReturnRecovery	DaysRecovery	ReturnDecline	ReturnRecovery	DaysRecovery
	(1)	(2)	(3)	(4)	(5)	(6)
LossCarryback#TaxRate	0.8683***	1.4528	− 1907.7531	0.4098	− 0.2053	− 821.6534
	(3.13)	(0.86)	(− 1.62)	(1.59)	(− 0.23)	(− 0.97)
LossCarryforward#TaxRate	− 0.3239	− 4.4298***	626.9036	− 0.4912	− 1.0830	452.7445
	(− 0.99)	(− 3.40)	(0.50)	(− 1.30)	(− 1.09)	(0.30)
LossCarryback	− 0.1979***	− 0.1355	299.8713	− 0.0753	0.1087	142.7345
	(− 2.60)	(− 0.30)	(0.89)	(− 1.12)	(0.49)	(0.65)
LossCarryforward	0.1179	1.3055***	− 339.4381	0.1638	0.3634	− 219.3884
	(1.25)	(3.45)	(− 1.00)	(1.55)	(1.33)	(− 0.56)
GroupTaxSystem	− 0.0019	0.0016	− 104.6643*	0.0174	− 0.0081	− 45.8333
	(− 0.15)	(0.03)	(− 1.67)	(1.16)	(− 0.26)	(− 0.84)
TaxRate	− 0.5946***	0.5194	1072.9689**	− 0.2940	− 0.4829	953.5512
	(− 3.68)	(0.60)	(1.98)	(− 1.50)	(− 0.98)	(1.53)
CompanyRisk				− 0.0002**	0.0001	0.7037*
				(− 2.02)	(0.18)	(1.93)
Controls	Yes	Yes	Yes	Yes	Yes	Yes
Crisis FE	Yes	Yes	Yes	Yes	Yes	Yes
Industry FE	Yes	Yes	Yes	Yes	Yes	Yes
Crisis− Industry FE	Yes	Yes	Yes	No	No	No
Observations	3,139	2,729	2,126	1,056	1,028	784
Centered R^2^	0.1447	0.0933	0.1132	0.1881	0.0536	0.0854

This table represents the robustness tests for results reported in Table 6. In Columns 1 to 3, we additionally use crisis−  industry fixed effects and Columns 4 to 6 present the results for additionally controlling for *CompanyRisk*. Data from the International Monetary Fund, EY Worldwide Corporate Tax Guide, and Thomson Reuters. The observational units are firms. See Appendix Table 16 for variable definitions. Heteroskedasticity-robust Driscoll-Kraay standard errors with multi-way clustering on year-industry-level and country-level in parentheses. *Indicates significance at the 10% level, **indicates significance at the 5% level, ***indicates significance at the 1% level

**Table 24:** Robustness tests II Hypothesis 3

Dependent Variable	Additional FEs						CompanyRisk					
	ReturnDecline	ReturnDecline	ReturnRecovery	ReturnRecovery	DaysRecovery	DaysRecovery	ReturnDecline	ReturnDecline	ReturnRecovery	ReturnRecovery	DaysRecovery	DaysRecovery
	Loss	Profit	Loss	Profit	Loss	Profit	Loss	Profit	Loss	Profit	Loss	Profit
LossCarryback	0.1224**	0.0229*	0.3046*	0.2471***	109.5154	− 221.2846***	0.0555	0.0187	0.4733***	0.0538	144.0128***	− 82.0352*
	(2.25)	(1.94)	(1.72)	(6.00)	(0.29)	(− 3.01)	(0.51)	(1.51)	(6.15)	(1.41)	(3.99)	(− 1.65)
LossCarryforward	0.1073	0.0095	0.2742	− 0.0725	100.1784	− 89.1434	0.1983*	0.0227	0.5423**	0.0547	− 4.4792	− 100.3065*
	(1.39)	(0.60)	(1.18)	(− 1.13)	(0.37)	(− 1.52)	(1.81)	(1.44)	(2.69)	(1.32)	(− 0.15)	(− 1.77)
GroupTaxSystem	0.1295*	0.0329	0.1382	0.2546**	− 400.3051*	− 325.2444***	0.0801	0.0168	0.3899*	0.0041	− 1721.9333***	− 94.2047
	(1.87)	(1.35)	(0.45)	(2.28)	(− 1.69)	(− 3.16)	(0.39)	(0.78)	(1.75)	(0.10)	(− 8.68)	(− 1.33)
TaxRate	0.2723	− 0.2194*	− 0.8381	1.4394***	− 1484.2236	− 172.9733	− 1.2341	− 0.2729*	2.4875	− 0.5568	0.0000	610.7149
	(0.30)	(− 1.89)	(− 0.39)	(3.28)	(− 0.91)	(− 0.39)	(− 0.83)	(− 1.94)	(1.00)	(− 1.47)	(0.00)	(1.35)
CompanyRisk							0.0009	− 0.0003**	0.0019	0.0001	0.3467	0.7362**
							(1.25)	(− 2.42)	(1.35)	(0.29)	(0.78)	(2.00)
*F−Test*												
LCB=LCF	0.8385	0.4805	0.9090	0.0000***	0.9686	0.0224**	0.3088	0.8305	0.7053	0.9851	0.0042***	0.6985
Crisis FE	Yes	Yes	Yes	Yes	Yes	Yes	Yes	Yes	Yes	Yes	Yes	Yes
Industry FE	Yes	Yes	Yes	Yes	Yes	Yes	Yes	Yes	Yes	Yes	Yes	Yes
Crisis− Industry FE	Yes	Yes	Yes	Yes	Yes	Yes	No	No	No	No	No	No
Observations	227	2,772	186	2,403	120	1,877	58	982	56	957	41	731
Centered R^2^	0.3374	0.1550	0.0653	0.1359	0.3768	0.2093	0.5650	0.2031	0.6229	0.0577	0.9366	0.0964

This table represents the robustness tests for results reported in Table 7. In Columns 1 to 6, we additionally use crisis-industry fixed effects and Columns 7 to 12 present the results for additionally controlling for *CompanyRisk*. Data from the International Monetary Fund, EY Worldwide Corporate Tax Guide, and Thomson Reuters. The observational units are firms. See Appendix Table 16 for variable definitions. Heteroskedasticity-robust Driscoll-Kraay standard errors with multi-way clustering on year-industry-level and country-level in parentheses. *Indicates significance at the 10% level, **indicates significance at the 5% level, ***indicates significance at the 1% level

**Table 25:** Separate crisis effects Hypothesis 1

Dependent Variable	2008 Crisis			2020 Crisis		
	ReturnDecline	ReturnRecovery	DaysRecovery	ReturnDecline	ReturnRecovery	DaysRecovery
	(1)	(2)	(3)	(4)	(5)	(6)
LossCarryback	0.1102***	0.3333***	− 216.8134	− 0.0023	0.0670***	− 23.0164
	(3.45)	(2.62)	(− 1.34)	(− 0.15)	(2.63)	(− 0.95)
LossCarryforward	0.0798***	0.2538**	− 346.6853**	0.0107	0.0632*	− 17.2163
	(2.76)	(2.03)	(− 2.34)	(0.53)	(1.93)	(− 0.59)
GroupTaxSystem	− 0.0015	0.1094	− 811.4143**	− 0.0038	− 0.0128	29.1672
	(− 0.04)	(0.64)	(− 2.33)	(− 0.31)	(− 0.60)	(1.34)
TaxRate	− 0.0635	− 2.8588	2277.3370	− 0.8798***	− 1.5247***	541.8838**
	(− 0.11)	(− 1.29)	(0.77)	(− 5.86)	(− 5.97)	(2.31)
Industry FE	Yes	Yes	Yes	Yes	Yes	Yes
Observations	903	903	608	2,236	1,826	1,517
Centered R^2^	0.2733	0.2181	0.3216	0.2395	0.1361	0.0980

This table represents the results for our baseline specification for the two investigated crises separately. Data from the International Monetary Fund, EY Worldwide Corporate Tax Guide, and Thomson Reuters. The observational units are firms. See Appendix Table 16 for variable definitions. Heteroskedasticity-robust Driscoll-Kraay standard errors with multi-way clustering on year-industry-level and country-level in parentheses. *Indicates significance at the 10% level, **indicates significance at the 5% level, ***indicates significance at the 1% level

**Table 26:** Separate crisis effects Hypothesis 2

Dependent Variable	2008 Crisis			2020 Crisis		
	ReturnDecline	ReturnRecovery	DaysRecovery	ReturnDecline	ReturnRecovery	DaysRecovery
	(1)	(2)	(3)	(4)	(5)	(6)
LossCarryback#TaxRate	1.2415	− 4.4957	3980.0162	0.6348***	1.7307***	− 738.1405*
	(1.61)	(− 1.55)	(0.71)	(2.80)	(4.15)	(− 1.93)
LossCarryforward#TaxRate	0.7261	1.1214	− 3375.6441	− 0.7150*	0.5917	− 315.6325
	(0.69)	(0.24)	(− 0.55)	(− 1.67)	(0.81)	(− 0.52)
LossCarryback	− 0.2431	1.5994*	− 1332.1629	− 0.1625***	− 0.3735***	165.6455
	(− 1.08)	(1.83)	(− 0.88)	(− 2.68)	(− 3.39)	(1.57)
LossCarryforward	− 0.1307	− 0.0795	633.4669	0.2010*	− 0.0915	63.0147
	(− 0.42)	(− 0.06)	(0.36)	(1.73)	(− 0.46)	(0.37)
GroupTaxSystem	− 0.0292	0.2068	− 887.7023**	− 0.0040	0.0103	15.9822
	(− 0.60)	(1.28)	(− 2.00)	(− 0.30)	(0.41)	(0.69)
TaxRate	− 0.5564	− 2.4138	2888.9459	− 0.8347***	− 1.9618***	772.6927***
	(− 0.77)	(− 0.83)	(0.65)	(− 4.80)	(− 6.20)	(2.72)
Industry FE	Yes	Yes	Yes	Yes	Yes	Yes
Observations	903	903	608	2,236	1,826	1,517
Centered R^2^	0.2807	0.2202	0.3283	0.2485	0.1464	0.1063

This table represents the results for Hypothesis 2 for the two investigated crises separately. Data from the International Monetary Fund, EY Worldwide Corporate Tax Guide, and ThomsonReuters. The observational units are firms. See Appendix Table 16 for variable definitions. Heteroskedasticity-robust Driscoll-Kraay standard errors with multi-way clustering on year-industry-level and country-level in parentheses. *Indicates significance at the 10% level, **indicates significance at the 5% level, ***indicates significance at the 1% level

**Table 27:** Separate crisis effects Hypothesis 3

Dependent Variable	2008 Crisis					
	ReturnDecline		ReturnRecovery		DaysRecovery	
	Loss	Profit	Loss	Profit	Loss	Profit
LossCarryback	− 0.0931	0.1008***	− 0.2231	0.3415***	775.7003	− 191.0168
	(− 0.71)	(3.11)	(− 0.00)	(2.59)	(1.45)	(− 1.15)
LossCarryforward	0.4323***	0.0675**	1.3921	0.2159*	− 260.1556	− 388.3436***
	(4.00)	(2.35)	(0.01)	(1.67)	(− 0.26)	(− 2.74)
GroupTaxSystem	− 0.4053	− 0.0002	0.8821	0.1277	0.0000	− 782.1368**
	(− 1.20)	(− 0.01)	(0.01)	(0.72)	(0.00)	(− 2.22)
TaxRate	0.0000	− 0.1292	0.0000	− 2.8848	0.0000	2930.6960
	(0.00)	(− 0.23)	(0.00)	(− 1.25)	(0.00)	(0.99)
*F−Test*						
LCB=LCF	0.0131**	0.4566	0.9942	0.4888	0.2736	0.2290
Controls	Yes	Yes	Yes	Yes	Yes	Yes
Crisis & Industry FE	Yes	Yes	Yes	Yes	Yes	Yes
Observations	64	834	64	834	35	568
Centered R^2^	0.5076	0.2620	0.1208	0.2307	0.5158	0.3237

Dependent Variable	2020 Crisis					
	ReturnDecline		ReturnRecovery		DaysRecovery	
	Loss	Profit	Loss	Profit	Loss	Profit
LossCarryback	0.1700**	− 0.0073	0.2336*	0.0531**	− 289.7751***	− 16.7883
	(2.31)	(− 0.49)	(1.70)	(2.15)	(− 3.73)	(− 0.69)
LossCarryforward	0.2265**	− 0.0001	0.6439***	0.0250	− 730.1793***	− 10.6320
	(2.30)	(− 0.00)	(3.13)	(0.75)	(− 6.87)	(− 0.36)
GroupTaxSystem	0.1433**	− 0.0117	0.2343**	− 0.0185	183.6994***	27.8004
	(2.40)	(− 0.97)	(1.99)	(− 0.82)	(2.99)	(1.21)
TaxRate	− 1.7338*	− 0.8270***	− 3.6900**	− 1.3515***	4713.0582***	543.7918**
	(− 1.74)	(− 5.42)	(− 2.04)	(− 5.27)	(4.87)	(2.28)
*F−Test*						
LCB=LCF	0.4498	0.7277	0.0150**	0.3887	0.0002***	0.8059
Controls	Yes	Yes	Yes	Yes	Yes	Yes
Crisis & Industry FE	Yes	Yes	Yes	Yes	Yes	Yes
Observations	186	2,048	145	1,679	107	1,407
Centered R^2^	0.3176	0.2329	0.3103	0.1394	0.5642	0.0984

This table represents the results for Hypothesis 3 for the two investigated crises separately. Data from the International Monetary Fund, EY Worldwide Corporate Tax Guide, and Thomson Reuters. The observational units are firms. See Appendix Table 16 for variable definitions. Heteroskedasticity-robust Driscoll-Kraay standard errors with multi-way clustering on year-industry-level and country-level in parentheses. *Indicates significance at the 10% level, **indicates significance at the 5% level, ***indicates significance at the 1% level.

## Footnotes

1 Another argument for using home country tax rules is that -in the absence of country-by-country reporting -investors do not know the cross-country income distribution and can, therefore, hardly consider it.

2 We do not believe that the effects of the crisis, including the implications of tax loss offset regulations, are anticipated ex-ante. They should, therefore, not be reflected in pre-crisis stock prices. Hereby, we rely on the assumption that economic crises mainly come as unexpected events.

3 Other papers find stock price reactions to the recent US tax reform (see Koutney and Mills 2018; Wagner et al. 2018).

4 A google trend analysis can confirm this latter assumption of particular awareness. The keyword *loss carryback* received particular monthly attention during the past two economic crises (March 2009/April 2010: highest values over the whole period 2004 to 2022; April 2020: highest value since 2013). The keyword *Verlustrücktrag* received the highest monthly attention since 2004 in February 2021.

5 We rely on Horta et al. (2014) for the beginning and end of the 2008 recession. For the 2020 crisis, we consider the WHO reports on the first COVID-19 cases as starting point and finish our data collection in April 2021.

6 Since we focus on very large multinationals, we assume that a loss carryback or a loss carryforward that is restricted in amount is mainly ineffective for firms in our sample. Loss carryforward and loss carryback regulations of our sample countries differ primarily with regard to time restrictions. Most of our sample countries with a loss carryback restrict it to one year, while a loss carryback of two or three years is granted only in very few countries. Given this small variation, we disregard time restrictions for our baseline definition of LossCarryback. Contrastingly, time restrictions for loss carryforward regulations are much more relevant and differ between five years and no time restriction. Our results are not sensitive to the particular definition of our loss offset variables, as we document in the robustness test section.

7 Otherwise, firms from Japan (Nikkei 225), e.g., would implicitly enter the regression with 7.5 times the weight of US (Dow Jones 30) or German firms (Dax 30).

8 We document in a robustness test using an unweighted panel that our results are not driven to any considerable extent by the applied weighting mechanism.

9 The firm-individual value of a loss carryback depends on the marginal tax rate of the respective firm. Given that we investigate corporations with linear income tax schedules, this simplification is appropriate.

10 Excluding all firms with a negative pre-crisis stock market performance from our data ensures, to a certain extent, that both subsamples are comparable in economic terms.

11 Appendix Table 15 gives an overview of the sample countries and their respective indices. We could not include Argentina, Italy, South Korea, and Romania due to missing data in Thomson Reuters.

12 We thank EY Germany for providing us with the relevant materials.

13 In addition to the low correlation, we test the inter-correlation among the loss offset variables and find no redundancy of the three variables (Cronbach’s alpha 0.56).

14 Since this measure is a count variable, we alternatively use a Poisson model. Results are untabulated but show similar inferences.

15 The difference is statistically significant at the ten percent confidence level only in the case of specification (2).

16 See Appendix Table 19 for a detailed description of determining *Combined2*.

17 Appendix Table 15 shows a list of these countries.

18 We would like to highlight that the heterogeneous effects of loss carryforward and loss carryback in the two subsamples cannot be explained by general differences in profitability. These differences explain that the effects of both loss offset regulations are stronger in the loss-making subsample. They should, however, have no effect on the relative impact of loss carryforward compared to loss carryback.

19 We exclude companies from India, Indonesia, and Turkey for the 2008 crisis and Brazil, Greece, South Africa, and Turkey for the 2020 crisis from our sample.

20 The reduced sample size explains that statistical inferences are somewhat weaker here.

21 The first placebo crisis, thus, goes from 01.07.2012 to 30.06.2014, whereas the last placebo crisis goes from 01.01.2016 to 31.12.2017.

22 In contrast to Eq. 1, we cannot include year fixed effects since our placebo tests cover only one placebo crisis each. Instead, we cluster standard errors on industry-and country-level.
